# Spatiotemporal profile of an optimal host response to virus infection in the primate central nervous system

**DOI:** 10.1371/journal.ppat.1012530

**Published:** 2025-01-22

**Authors:** Olga A. Maximova, Sarah L. Anzick, Daniel E. Sturdevant, Richard S. Bennett, Lawrence J. Faucette, Marisa St. Claire, Stephen S. Whitehead, Kishore Kanakabandi, Zong-mei Sheng, Yongli Xiao, John C. Kash, Jeffery K. Taubenberger, Craig Martens, Jeffrey I. Cohen

**Affiliations:** 1 Laboratory of Infectious Diseases, National Institute of Allergy and Infectious Diseases, National Institutes of Health; Bethesda, Maryland, United States of America; 2 Rocky Mountain Laboratories, Research Technologies Branch, Genomics Research Section, National Institute of Allergy and Infectious Diseases, National Institutes of Health; Hamilton, Montana, United States of America; 3 Infectious Disease Pathogenesis Section, Comparative Medicine Branch, National Institute of Allergy and Infectious Diseases, National Institutes of Health; Bethesda, Maryland, United States of America; 4 Bioqual, Inc., Rockville, Maryland, United States of America; University of Pittsburgh, UNITED STATES OF AMERICA

## Abstract

Viral infections of the central nervous system (CNS) are a major cause of morbidity largely due to lack of prevention and inadequate treatments. While mortality from viral CNS infections is significant, nearly two thirds of the patients survive. Thus, it is important to understand how the human CNS can successfully control virus infection and recover. Since it is not possible to study the human CNS throughout the course of viral infection at the cellular level, here we analyzed a non-lethal viral infection in the CNS of nonhuman primates (NHPs). We inoculated NHPs intracerebrally with a high dose of La Crosse virus (LACV), a bunyavirus that can infect neurons and cause encephalitis primarily in children, but with a very low (≤ 1%) mortality rate. To profile the CNS response to LACV infection, we used an integrative approach that was based on comprehensive analyses of (i) spatiotemporal dynamics of virus replication, (ii) identification of types of infected neurons, (iii) spatiotemporal transcriptomics, and (iv) morphological and functional changes in CNS intrinsic and extrinsic cells. We identified the location, timing, and functional repertoire of optimal transcriptional and translational regulation of the primate CNS in response to virus infection of neurons. These CNS responses involved a well-coordinated spatiotemporal interplay between astrocytes, lymphocytes, microglia, and CNS-border macrophages. Our findings suggest a multifaceted program governing an optimal CNS response to virus infection with specific events coordinated in space and time. This allowed the CNS to successfully control the infection by rapidly clearing the virus from infected neurons, mitigate damage to neurophysiology, activate and terminate immune responses in a timely manner, resolve inflammation, restore homeostasis, and initiate tissue repair. An increased understanding of these processes may provide new therapeutic opportunities to improve outcomes of viral CNS diseases in humans.

## Author summary

Viral infections of the brain and spinal cord can be devastating and even fatal in some patients, while others survive and recover. Understanding how the human brain and spinal cord can successfully control virus infection may lead to improved treatments and help to determine the optimal timing for their use. Here, we studied how the brain and spinal cord of nonhuman primates respond to virus infection after inoculation of La Crosse virus directly into the brain. We identified the location, timing, and functional repertoire of optimal host responses in the primate brain and spinal cord that led to rapid virus clearance from infected neurons. These host responses were carefully regulated at the levels of gene expression and cellular functions to mitigate neurological damage, resolve inflammation, restore homeostasis, and initiate tissue repair. A better understanding of the processes that govern optimal responses to viral infection in the brain and spinal cord may improve outcomes of viral neurological diseases and allow full recovery in a higher proportion of patients.

## Introduction

Despite an increasing understanding of the pathogenesis of viral infections of the central nervous system (CNS) (reviewed in [1–5]), treatments for most of these infections remain largely supportive. However, while the mortality from viral infections of the CNS in humans can be significant, nearly two thirds of the patients survive [6], although often with neurological sequelae. Moreover, many individuals have asymptomatic infection or mild CNS disease that is not diagnosed since they do not seek medical care. For example, CNS disease develops in less than 1% of human infections with West Nile virus (WNV), mostly in patients with advanced age, comorbidities, or immunodeficiencies [7]. A better understanding of physiological processes that allow the human CNS to successfully control neuroinvasive viral infections and recover may provide new therapeutic approaches to improve outcomes of these infections. However, it is difficult to study the human CNS at the cellular and molecular levels since obtaining brain tissue from living persons is usually limited to a single time point at best (often reporting a non-specific diagnosis of “encephalitis” [8]), while autopsy can only provide CNS tissue from patients with an unsuccessful outcome.

To elucidate attributes of successful CNS responses to viral infections that result in full recovery and can be reasonably extrapolated to humans, we modeled a CNS infection longitudinally using nonhuman primates (NHPs) intracerebrally inoculated with a neuropathogenic but non-lethal virus. The intracerebral NHP model has been valuable for elucidating many aspects of viral pathogenesis within the CNS as well as for testing the safety of vaccines [9–14]. In the present study we used La Crosse virus (LACV), a mosquito-borne bunyavirus that is a leading cause of viral encephalitis in pediatric populations, but with very low (≤ 1%) mortality and high recovery rates [15,16]. While most knowledge of LACV pathogenesis in the CNS at the cellular level has been obtained from experiments in mice [17–22], relevant information from humans is very scarce. At present, only general pathological findings are available for human brain tissue and only from three pediatric cases of LACV encephalitis (postmortem tissue was analyzed from two cases [23] and biopsy tissue was examined in one case [24]). The pathological changes in the postmortem tissue were non-specific and included inflammatory and neurodegenerative changes in the cerebral cortex, basal ganglia, and brainstem [23]. The biopsy report identified neurons as the cell type infected by LACV [24].

Here, we have profiled the host responses in the CNS of NHPs after intracerebral inoculation with LACV using an integrative approach that was based on comprehensive analyses of (i) spatiotemporal dynamics of virus replication, (ii) identification of types of infected neurons, (iii) spatiotemporal transcriptomics, and (iv) morphological and functional changes in CNS-intrinsic and extrinsic cells. We found that the primate CNS can successfully clear the virus from infected neurons, mitigate tissue damage, resolve inflammation, restore homeostasis, and initiate repair.

## Results

### Experimental LACV infection of the CNS in nonhuman primates is confined to neurons of the brain, spares the spinal cord, and is cleared within three weeks

**Fig 1:**
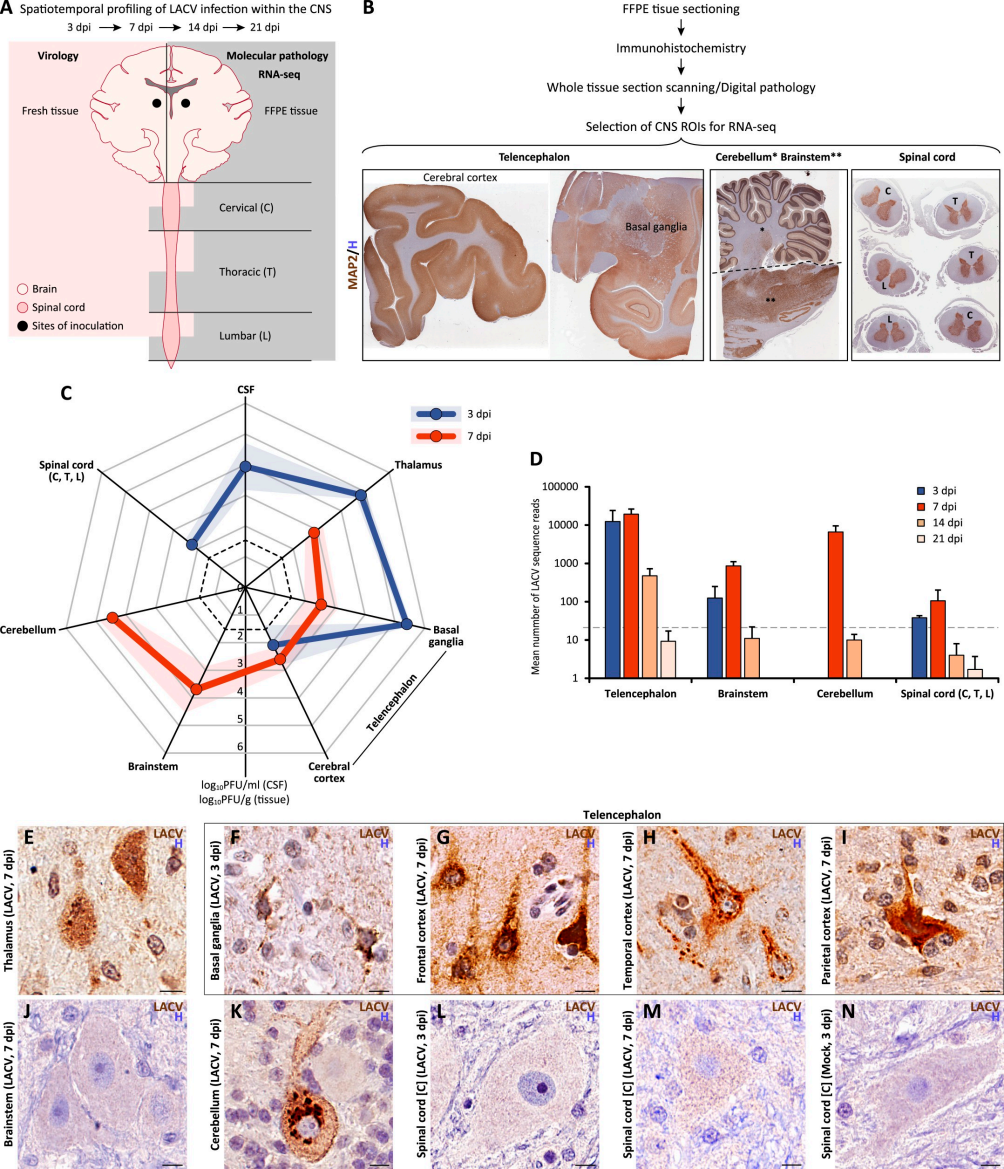
Spatiotemporal profile of virus infection in the CNS of NHPs intrathalamically inoculated with LACV. (**A** and **B**) Integrative design shows the timing, tissue samples, and downstream analyses used in this study. Abbreviations for the spinal cord regions in B are indicated in A. (**C**) Radar graph shows mean LACV titers (± standard errors; shaded areas) in the CNS regions of interest (ROIs) at indicated dpi. Dashed line indicates the limit of detection (1.7 log10 PFU). Data on LACV titers in the CNS of each individual NHP (from two NHPs per time point) are provided in S2 Fig. (**D**) Mean number of LACV sequence reads (± SE) per 100 ng of RNA detected by RNA-seq in the FFPE tissue in indicated CNS ROIs of LACV-infected NHPs at indicated dpi. Dashed line shows the background threshold based on the three times the number of reads in mock at 3 dpi (n = 21). (**E—N**) Representative cellular immunoreactivity for LACV Gc glycoprotein (LACV, brown) with hematoxylin counterstaining (H, blue) in indicated CNS ROIs of LACV-infected NHPs (E—M) and one representative mock (N) at indicated dpi. Scale bars (E—N): 10 μm.

To study the pathogenesis of LACV infection in the CNS, eight young LACV-seronegative rhesus monkeys (ages 2–3 years old) were inoculated in the thalamus bilaterally with LACV/human/1978/WI [25] (hereafter LACV) at a total dose of 5 log_10_ plaque forming units (PFU). Four LACV-seronegative rhesus monkeys of the same age were inoculated in the thalamus bilaterally with cell culture medium lacking virus as controls (hereafter mock). All animals were monitored for neurological signs and three animals (LACV-infected, n = 2; mock, n = 1) were euthanized at each of the pre-determined time points: 3, 7, 14, and 21 days post inoculation (dpi). None of the LACV-inoculated or mock animals developed detectable viremia or any overt neurological signs during the observation period. However, all LACV-inoculated animals became infected with the virus since rising virus neutralizing antibody titers were detected in the serum from 10 dpi onward and in the cerebrospinal fluid (CSF) at 21 dpi (S1 Table). This indicates that although the animals had no overt signs of neurological disease, LACV infection of the CNS was established in all inoculated NHPs. The integrative design used in this study is summarized in Fig 1A and 1B and a detailed overview of the CNS regions of interest (ROIs) analyzed in this study by molecular pathology and RNA-seq is given in S1 Fig. More details on the neuroanatomical tissue dissection and digital pathology workflow are provided in the Materials and Methods.

To understand how the CNS of NHPs controlled LACV infection, we analyzed: (i) the spatiotemporal profile of virus infection in the telencephalon (cerebral cortex and basal ganglia), brainstem (midbrain, pons, and medulla oblongata), cerebellum, and spinal cord (cervical, thoracic, and lumbar regions) based on virus titration in cell culture (virology) using fresh tissue samples (Fig 1A; shown in pink on the left) and detection of the LACV RNA by RNA-sequencing (RNA-seq) using formalin fixed paraffin embedded (FFPE) tissue sections from the contralateral brain hemisphere (Fig 1A; shown in gray and expanded in Fig 1B); and (ii) the type of cells infected with LACV based on detection of LACV Gc glycoprotein by immunohistochemistry.

The virus was detected in the cerebrospinal fluid (CSF) only at the earliest time point (3 dpi) and not thereafter (see Fig 1C for mean virus titers and S2 Fig for virus titers in individual NHPs). The CSF virus titer at that time was 1 log_10_ PFU/ml lower than that of the inoculum (i.e., 4 log_10_ PFU/ml versus 5 log_10_ PFU/ml, respectively). Assuming that the virus inoculum dilution factor was 1:10, based on the approximately 10 ml estimated volume of the CSF in rhesus macaques [26], this suggests that most of the CSF virus at this early time point after inoculation was derived from the injected inoculum leaking into the interstitial fluid (ISF) and CSF. Thus, we used the CSF virus titer at 3 dpi (4 log_10_ PFU/ml) as a cut-off value, above which virus titers in the CNS ROIs would be indicative of productive virus replication in the CNS parenchyma, rather than being inoculum-derived input to the tissue samples from the ISF/CSF. Based on this criterion, at 3 dpi LACV replication was limited to the thalamus and basal ganglia (the site of inoculation and adjacent area, respectively) (Fig 1C; 3 dpi: blue line). At 7 dpi, virus was no longer detected in the CSF, indicating that any virus detected at this time point should be due to replication in the cells of the CNS parenchyma. Thus, one week after intrathalamic inoculation, LACV replicated in the cells of the thalamus, telencephalon (i.e., cerebral cortex and basal ganglia), brainstem, and cerebellum (Fig 1C; 7 dpi: red line). No virus replication was detected in any region of the spinal cord at 7 dpi. At 14 dpi, a very low virus titer (1.9 log_10_ PFU/g, just above the limit of detection [LOD] of 1.7 log_10_ PFU/g) was detected in the temporal cortex of one NHP (S2C Fig) but the mean virus titer from all samples of the cerebral cortex (i.e., frontal, temporal, parietal, and occipital cortices) was below the LOD. No infectious virus was detected at 21 dpi in any of the CNS ROIs.

We also detected LACV RNA sequences in the CNS ROIs using RNA-seq (Fig 1D). As expected, RNA-seq was more sensitive than virus titration in cell culture. RNA-seq detected 7 LACV RNA sequence reads in the cerebral cortex from one mock monkey at 3 dpi. Since this low number of LACV RNA sequence reads likely represents contamination during storage and/or handling of the FFPE tissue block, we tripled this number and set it as a background threshold (n = 21). At day 3, most LACV sequences were detected in the telencephalon (encompassing the basal ganglia and cerebral cortex, the areas adjacent to inoculation site). At day 7, LACV sequences were detected in all CNS ROIs. At day 14, the number of LACV sequences exceeded that of background threshold only in the telencephalon. At day 21, a few LACV sequences were detected, but they were below the threshold established as background. Thus, LACV RNA spatiotemporal profiling, while being more sensitive, was generally consistent with the virus titers. Since LACV titers, unlike viral RNA sequences, indicate the presence of replicating virus, we can conclude that infectious LACV was cleared from all CNS ROIs by the third week after inoculation.

To determine the cell types in the CNS that supported LACV replication, we performed immunohistochemistry using an antibody to LACV Gc glycoprotein. In all CNS ROIs, LACV Gc glycoprotein was detected only during the first week of infection (3 and 7 dpi) exclusively in neurons of the thalamus (Fig 1E), telencephalon (Fig 1F–1I), and cerebellum (Fig 1K). LACV infected neurons in the basal ganglia (Fig 1F), pyramidal neurons in the frontal and temporal cortices (Fig 1G and 1H), corticospinal neurons (Betz cells) in the parietal cortex (Fig 1I), and Purkinje cells in the cerebellar cortex (Fig 1K). LACV Gc glycoprotein was absent from neurons of the brainstem (Fig 1J) and spinal cord (Fig 1L and 1M) of LACV-infected NHPs at any time point. No LACV Gc glycoprotein was detected in any CNS ROI-matched tissue of mock NHPs at any time point (example is given in Fig 1N). These findings demonstrate that LACV replicated in neurons within a week after infection; however, the infection was confined to neurons within and adjacent to the injection site (i.e., thalamus and basal ganglia) and to first/second order connected neurons (i.e., neurons in the cerebral and cerebellar cortices). Importantly, the absence of LACV Gc glycoprotein in spinal cord neurons is consistent with the failure of the virus to replicate in the spinal cord (Fig 1C), despite infection of the corticospinal neurons (Betz cells) that innervate the spinal cord neurons (Fig 1I). Taken together, these results demonstrate that the CNS of nonhuman primates blocked the spread of LACV beyond the brain (sparing the spinal cord) and had completely cleared the virus within three weeks of infection.

### The CNS responds to neuronal virus infection by downregulating neurophysiological processes proportionally to the damage inflicted

To study how LACV infection of neurons affects their function spatiotemporally, we first performed immunohistochemistry on FFPE tissue sections from all major CNS regions of the LACV-infected and mock animals for the microtubule associated protein 2 (MAP2) and analyzed the whole tissue sections using digital pathology. MAP2 immunoreactivity (MAP2-IR) delineated the somatodendritic compartments of all types of neurons. This facilitated neuroanatomical orientation using primate brain maps [27] and selection of the CNS ROIs and FFPE tissue blocks for RNA-seq. We selected the telencephalon, cerebellum, brainstem, and spinal cord (Figs 1B and S1). The diencephalon (containing the thalamus, the site of bilateral inoculations) and midbrain (containing the substantia nigra, present at the level of sections containing the thalamus) were excluded from RNA-seq to avoid potentially confounding artifacts from injection on gene expression.

**Fig 2:**
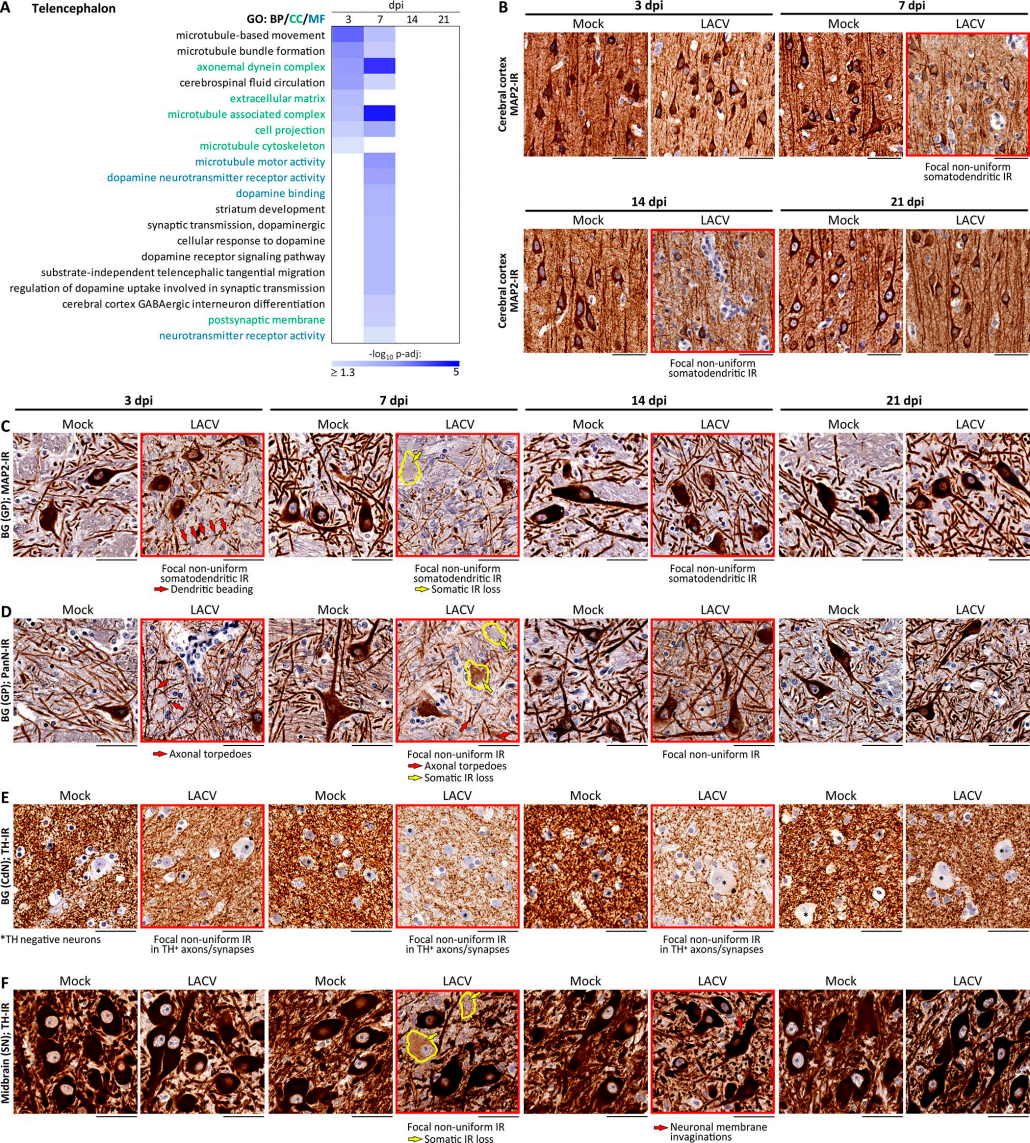
Time-limited downregulation of neurophysiological processes in the CNS of NHPs during LACV infection. (**A**) Temporal heatmap shows functional enrichment for transcriptional downregulation of the neurophysiological processes during 3 weeks after LACV inoculation. The color scale is based on the significance (negative log_10_ of the adjusted p-values [-log_10_ p-adj]). The gene ontology (GO) sources: BP, Biological Process; CC, Cellular Component; and MF, Molecular Function (terms are highlighted by respective colors). Lists of genes downregulated in the telencephalon at each dpi and data associated with (A) are provided in S1 File. **(B—F**) Transient focal changes in the cellular compartments of neurons in the telencephalon: (**B**) cerebral cortex; (**C**—**E**) basal ganglia (BG) (C and D, globus pallidus [GP]; E, caudate nucleus [CdN]); and (**F**) midbrain (substantia nigra [SN]) during LACV infection are revealed by the morphological changes in immunoreactivity (IR; brown with blue counterstaining) for indicated proteins. LACV panels show representative areas from two NHPs at indicated dpi side-by-side with the dpi-matched mock for comparisons. Supporting information that includes digital pathology analysis at increasing magnifications for both LACV-infected NHPs at each dpi is provided in S3 Fig. Indicated IRs reveal the following cellular compartments of neurons: MAP2-IR—somatodendritic compartments (B and C); PanN-IR—neuronal somata, dendrites, and axons (D); TH- (tyrosine hydroxylase)-IR—(i) excitatory projections (axons) and terminals (synapses) of nigral neurons (F) that innervate the TH negative BG neurons (E) and (ii) excitatory dopaminergic nigral neurons and their processes. Panels with indicated pathological changes in the neuronal cellular compartments are framed in red. Neurons with the somatic loss of indicated IR at 7 dpi are outlined in yellow in C, D, and F (tissue fields were chosen to include neighboring neurons with higher levels of respective IR to serve as internal control). Supporting information for corresponding changes in the protein IRs in dpi matched LACV-infected NHPs other than shown in D—F is provided in S4 Fig. Scale bars: 50 μm (B—F).

Bulk RNA-seq detected a transient transcriptional downregulation of neurophysiological processes at 3 and 7 dpi, with a return to baseline (defined as not significantly different from the dpi-matched mock; -log_10_ p-adj < 1.3) by 14 dpi (Fig 2A). At 3 and 7 dpi, there was significant downregulation of gene functions associated with CSF circulation in the telencephalon, suggesting an attempt by the host to limit virus spread within the CNS by this route. Importantly, the transient transcriptional dysregulation was largely restricted to the CNS ROIs with infected neurons (telencephalon [Fig 2A] and cerebellum [S5A Fig]) and affected microtubule-based processes, likely in the dendrites and axons. The telencephalon (ROI containing the cerebral cortex and basal ganglia which were adjacent to the intrathalamic virus inoculation zone) was affected earlier (at 3 dpi; Fig 2A) than the cerebellum (7 dpi; S5A Fig). By 7 dpi, transcriptional dysregulation in the telencephalon had expanded with disruption of the dopaminergic neurotransmission, receptors, uptake, and the cellular response to dopamine (Fig 2A; second column at 7 dpi). In contrast, the brainstem and spinal cord that had no detectable LACV-infected neurons, showed only transient transcriptional downregulation of biological processes related to tissue development and regulation of the extracellular matrix (S6A Fig). Thus, the CNS responded to LACV infection with a spatially restricted and time-limited transcriptional downregulation of neurophysiological processes in a manner that was strictly proportional to cellular damage, with a complete recovery of the transcriptional homeostasis by the second week after onset of infection.

To verify changes in transcriptional regulation of the neurophysiology at the levels of protein expression and cell morphology, we performed immunohistochemistry, focusing on spatiotemporal expression of proteins relevant to dysregulated neurophysiological processes identified by functional analysis of gene expression. To examine changes associated with the microtubule-based processes, we analyzed MAP2-IR (highly and uniformly expressed in the neuronal somata and dendrites under normal physiological conditions). In the cerebral cortex, somatic MAP2-IR remained high, but dendritic MAP2-IR became non-uniform at 7 and 14 dpi focally around the perivascular inflammatory cell infiltrates (Fig 2B and S3 Fig). In contrast, somatodendritic MAP2-IR decreased as early as at 3 dpi in the basal ganglia, with some neuronal dendrites exhibiting multiple varicosities arranged in beading patterns (Fig 2C; 3 dpi; LACV; compare to the dpi-matched mock). At 7 dpi, somatodendritic MAP2-IR in the basal ganglia had further decreased in the neuronal dendrites and became depleted in some neuronal somata (Fig 2C; 7 dpi; LACV; compare to the dpi-matched mock). At 14 dpi, MAP2-IR had recovered, and neuronal dendrites no longer displayed varicosities. By 21 dpi, MAP2-IR had completely recovered to the level of dpi-matched mock (Fig 2C; 21 dpi; LACV; compare to the dpi-matched mock).

To further dissect changes associated with cell projections (highly enriched CC term; Fig 2A), we performed immunohistochemistry on telencephalon sections with pan-neuronal antibodies (PanN), which identify axons in addition to neuronal somata and dendrites. At 3 dpi, PanN-IR revealed the presence of ovoid swellings (torpedoes) in some axons in the basal ganglia (globus pallidus) (Fig 2D; 3 dpi; LACV; compare to the dpi-matched mock). At 7 dpi, these axonal torpedoes persisted, and PanN-IR markedly decreased in neuronal somata, dendrites, and axons. At 14 dpi, PanN-IR increased, and by 21 dpi PanN-IR had completely recovered to the level of dpi-matched mock. Thus, the kinetics of PanN-IR (Fig 2D and S4A–S4D Fig) resembled that of MAP2-IR (Fig 2C).

Since transcriptional downregulation of dopaminergic neurotransmission was detected in the telencephalon at 7 dpi of LACV infection (Fig 2A), we examined the integrity of dopaminergic neurons in the substantia nigra (midbrain, a rostral part of the brainstem) (Fig 2F) and their dopaminergic projections to the basal ganglia (Fig 2E) by immunostaining for tyrosine hydroxylase (TH; a protein marker of dopaminergic neurons and projections). Similar to the kinetics seen with MAP2-IR and PanN-IR during LACV infection, there was a transient reduction of TH-IR in the somata of some substantia nigra neurons at 7 dpi (Fig 2F) and TH-IR associated with the dopaminergic projections (axons and synapses) of substantia nigra neurons in the caudate nucleus (part of the basal ganglia) became focally non-uniform compared to dpi-matched mock at 3, 7, and 14 dpi (Fig 2E). TH-IR had completely recovered in the somata of substantia nigra neurons and their projections in the basal ganglia to the level of dpi-matched mock controls by 21 dpi (Fig 2E and 2F; and S4E–S4H Fig). In addition, neuronal membrane invaginations seen in some substantia nigra neurons at 14 dpi had disappeared by 21 dpi (Fig 2F).

In the cerebellum, kinetics of transcriptional downregulation of neurophysiological processes during LACV infection mirrored that in the telencephalon, with predominant effects on microtubule-based processes and neurotransmission (S5A Fig). Immunohistochemistry verified these findings with non-uniform somatodendritic MAP2-IR at 3 and 7 dpi of LACV infection (S5F and S5G Fig). At 14 dpi, there was a very rare focal depletion of MAP2-IR in the Purkinje cell layer, granule cell layer, and partially in the molecular layer, which was strictly spatially associated with increased pial lymphocytic infiltration (S5H Fig). Similar to the telencephalon, MAP2-IR in the cerebellum returned to levels seen in mock controls by 21 dpi (S5I Fig). In contrast, the brainstem (pons and medulla) and spinal cord, apart from having a slight hypercellularity in the neuropil within first two weeks postinfection, maintained neuronal integrity at all time points, consistent with the lack of transcriptional downregulation of neurophysiological processes in these CNS ROIs (S6 Fig).

Taken together, these results show that the CNS responds to LACV infection of neurons by transient transcriptional downregulation of neural functions, which is associated with reduction in expression of relevant neuronal proteins and structural changes in neurons and their projections. The transient nature of changes in gene and protein expression, as well as resultant changes in the neuronal morphology is underscored by a complete return to homeostasis by the third week after onset of infection. Notably, there was a one-week lag in returning to homeostatic protein expression in neurons and their normal morphology, following the cessation of transcriptional downregulation of relevant biological processes. Collectively, the downregulation of neurophysiological processes in the CNS of NHPs during LACV infection appeared to be tightly spatiotemporally regulated, proportional to inflicted damage, and reversible.

### The CNS regulates transcriptional activation of host defense responses according to the extent of virus infection of neurons

**Fig 3:**
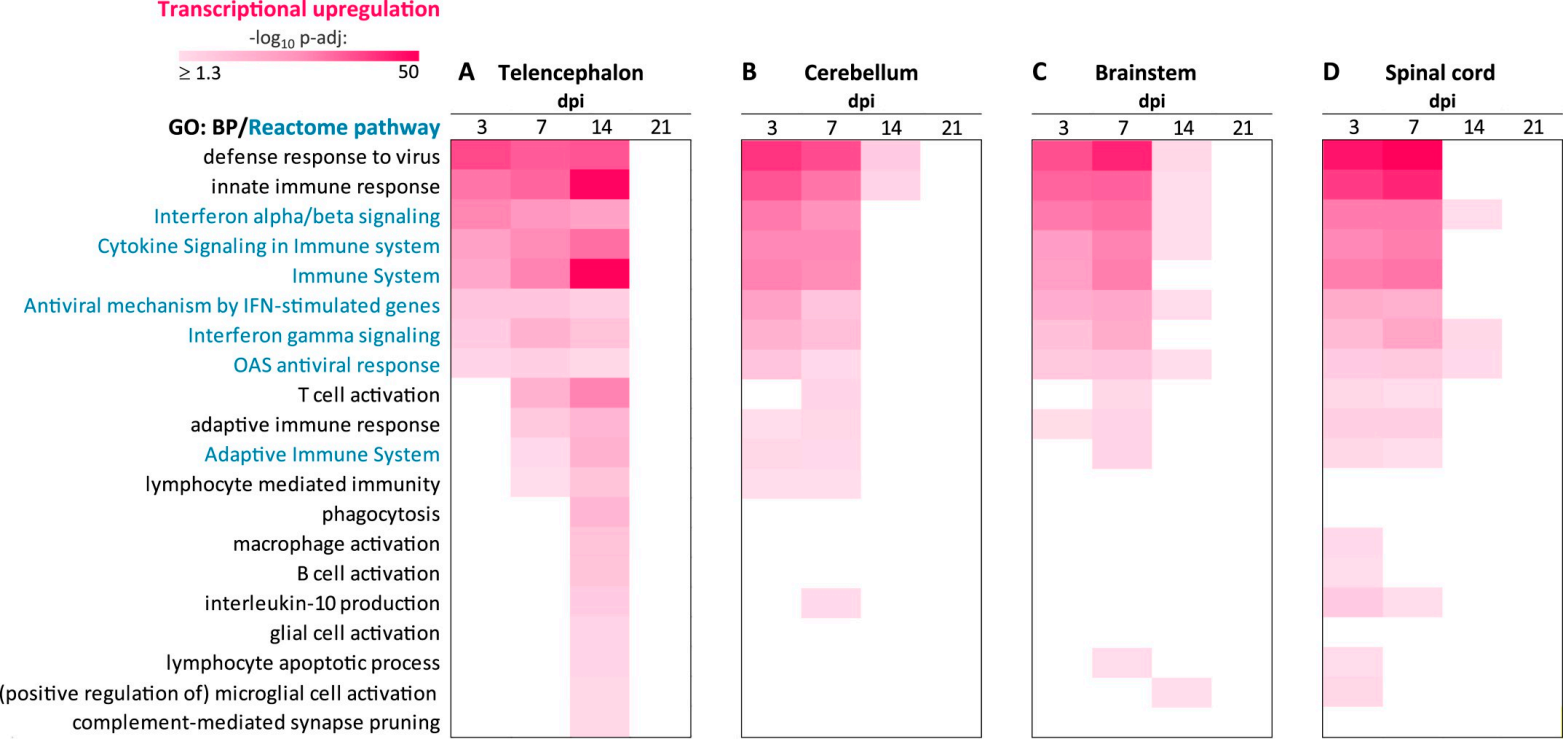
Transcriptional activation of host defense responses in the CNS of NHPs during LACV infection. (**A—D**) Temporal heatmaps show significant functional annotations based on genes that were upregulated (compared to the dpi-matched mock) in indicated CNS ROIs during LACV infection. The color scale is based on the level of significance and expressed as the negative log_10_ of the adjusted p-values (-log_10_ p-adj). The gene ontology sources GO: BP (Biological Process) and Reactome pathways were used and indicated by corresponding text colors. Top 20 manually collapsed significant genomic terms of interest are plotted for all comparisons. Lists of genes upregulated in the telencephalon, cerebellum, brainstem, and spinal cord at each dpi and data associated with (A—D) are provided in S2 File.

Next, we spatiotemporally profiled the transcriptional defense responses in the telencephalon, brainstem, cerebellum, and the spinal cord during LACV infection. Functional analysis of upregulated genes revealed rapid activation (detected at the earliest pre-determined time point, 3 dpi) of antiviral responses throughout in the CNS (Fig 3). These defense responses were activated not only in the CNS ROIs in which LACV actively replicated (i.e., telencephalon, brainstem, and cerebellum; Fig 1C) and where viral Gc glycoprotein was detected in neurons (i.e., telencephalon and cerebellum; Fig 1F–1I and 1K), but also in the spinal cord, which was spared from virus infection (Fig 1C, 1L, and 1M).

At 3 dpi, all CNS ROIs responded to LACV infection with robust transcriptional upregulation of innate immune processes and pathways, including activation of cytokine signaling (interferon alpha/beta/gamma), interferon-stimulated genes, and the OAS antiviral response (Fig 3A–D). Upregulation of these processes continued at 7 dpi in all CNS ROIs; however, in contrast to the telencephalon, where it persisted until 14 dpi (Fig 3A), upregulation became less significant after 7 dpi in the cerebellum (Fig 3B), brainstem (Fig 3C), and spinal cord (Fig 3D). Importantly, none of these processes was significantly upregulated at 21 dpi, suggesting that by this time transcriptional activation of the innate antiviral responses was terminated in all CNS ROIs.

In the telencephalon, the adaptive immune response was activated between 7 and 14 dpi but was no longer significant at 21 dpi. Notably, at the height of upregulated lymphocyte mediated immunity, the telencephalon showed upregulation of the lymphocyte apoptosis process (Fig 3A). Activation of the adaptive immune response was more variable, less significant, and shorter-lived (i.e., not significant past 7 dpi) in the cerebellum, brainstem, and spinal cord (Fig 3B–3D).

Taken together, these results show that after infection with LACV, transcriptional host defense responses were activated throughout the CNS, regardless of the site of virus replication. However, the timing, magnitude, and duration of these defense responses appeared to be adjusted to the extent of virus infection of neurons, while limiting unnecessary neuroinflammation in regions where the virus was no longer a threat. Importantly, regardless of the spatiotemporal variations during the first two weeks, transcriptional upregulation of biological processes governing antiviral defense responses was no longer detected in any of the CNS ROIs by the third week after infection onset.

### The CNS coordinates transcriptional responses to resolve neuroinflammation and restore homeostasis after virus infection is cleared

**Fig 4:**
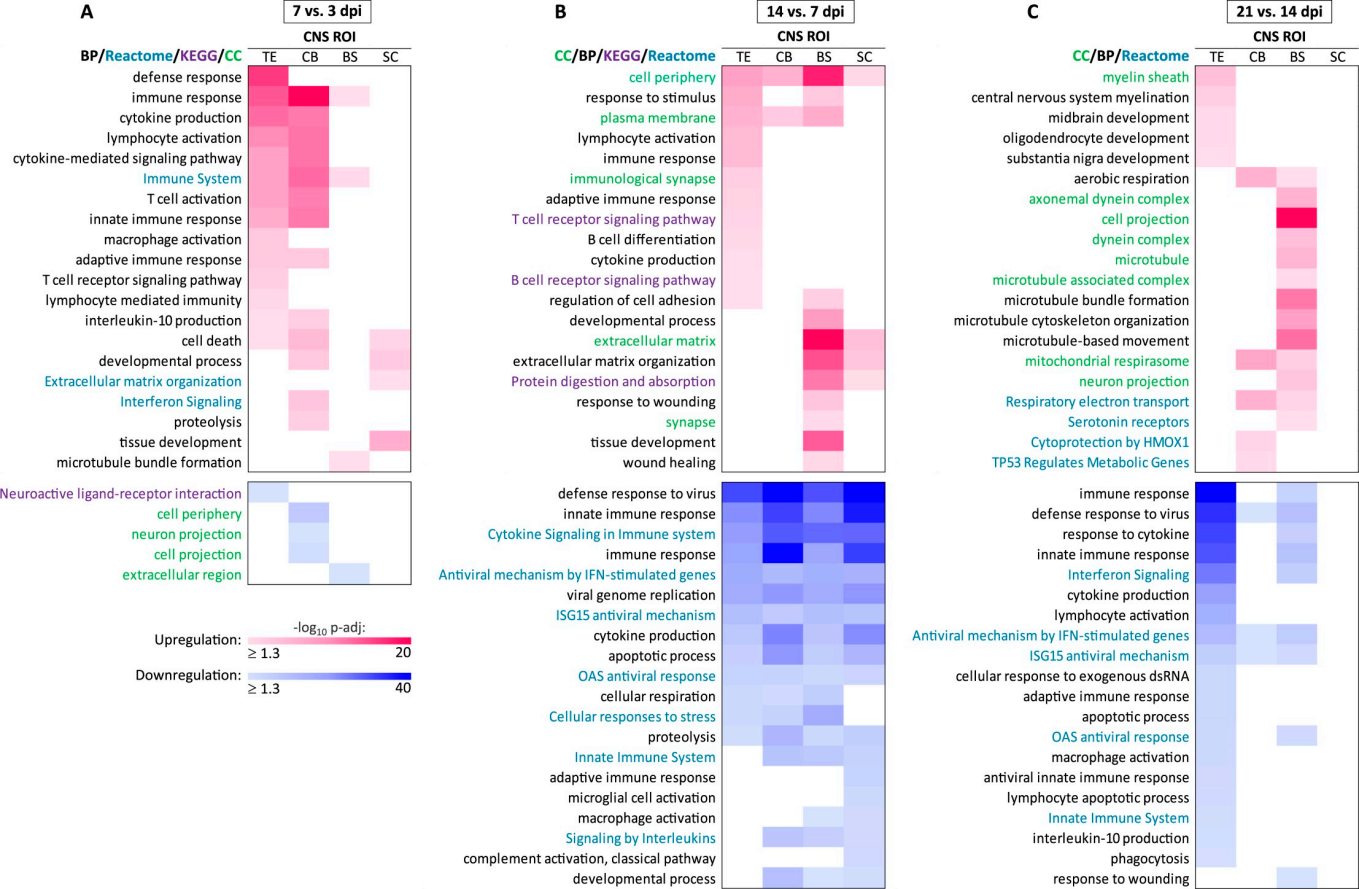
Time-differential transcriptional regulation of the CNS of NHPs during LACV infection. (**A—C**) Heatmaps show the time-differential (a time point of interest versus a preceding probed time point) functional changes in the transcriptional upregulation (red gradient) and downregulation (blue gradient) in four CNS ROIs (TE, telencephalon; CB, cerebellum; BS, brainstem; and SC, spinal cord). Differentially expressed genes were identified by comparing expression between the time points indicated above each heatmap. The color scales (-log_10_ p-adj) in (A) also apply to (B) and (C). Gene ontology sources are indicated in each heatmap by corresponding text colors. Top 20 manually collapsed significant genomic terms of interest are plotted for all comparisons, except for downregulation in (A), which was relatively limited. Lists of upregulated and downregulated genes analyzed in the telencephalon, cerebellum, brainstem, and spinal cord and data associated with (A—C) are provided in S3 File.

Next, we assessed whether the return to transcriptional homeostasis by 21 dpi after onset of virus infection was a consequence of termination of upregulation in gene expression (as determined by comparison to dpi-matched mock, Fig 3) or the result of complex transcriptional regulation of biological processes over time. We analyzed the time-specific differential transcriptional regulation in the CNS by comparing specific days after LACV inoculation with prior time points (Fig 4). We found a stepwise change in the CNS transcriptome that was coordinated across space (depending on the infection status of specific neurons in the CNS ROIs) and time (depending on whether the virus was present or eliminated).

Transcriptional upregulation of antiviral defense/innate immune responses increased by 7 dpi (versus 3 dpi), when the virus was replicating in neurons of the telencephalon and cerebellum (Fig 4A). The adaptive immune response was activated at 7 dpi, but only in the telencephalon and cerebellum. Upregulation of these host defense responses coincided with downregulation of neurophysiological processes (reinforcing the findings described above, Figs 2, S3, S4A–S4H, and S5).

By the second week (14 dpi), the transcriptome in all CNS ROIs underwent a dramatic downregulation of numerous biological processes related to antiviral defense/innate immune responses (Fig 4B). At the same time, adaptive immune responses (e.g., T and B cell receptor signaling pathways) were upregulated in the telencephalon, the site containing virus-infected neurons in many regions of the cerebral cortex and basal ganglia one week earlier (Fig 1F–1I). In contrast, the CNS ROIs lacking detectable infected neurons (i.e., brainstem and spinal cord) showed upregulation of processes related to the extracellular matrix, tissue development, wound healing, and neurotransmission (GO CC: synapse).

We found no significant changes in CNS gene expression at 21 dpi when samples from infected animals were compared to mock controls at the same time point (Figs 2A, S5A, S6A, and 3A–3D), indicating that transcriptional regulation in the CNS had returned to normal levels. To understand how transcriptional regulation had been restored, we compared the CNS transcriptome at 21 dpi with that at 14 dpi. During this period (Fig 4C), all initially upregulated host defense processes (e.g., innate and adaptive immune responses, apoptosis, and phagocytosis) continued to be further downregulated in all CNS ROIs, most significantly in the telencephalon. There was no differential downregulation between 21 and 14 dpi in the spinal cord. At the same time, comparison of 21 dpi with 14 dpi showed upregulation of tissue development and repair processes directed to restore the microtubule-based processes, myelination, and mitochondrial respiration. Differential upregulation of two additional pathways at 21 dpi may have contributed to resolution of acute neuroinflammation with restoration of the cellular homeostasis by 21 dpi: “Cytoprotection by HMOX1” [28–31] and “*TP53* Regulates metabolic genes” [32]. These regulatory pathways may suggest new therapeutic approaches to regulate neuroinflammation and improve the outcome of viral infections of the CNS.

### The CNS effectively coordinates functions of the intrinsic and extrinsic cells in response to virus infection

To determine how the CNS of NHPs responded to LACV infection of neurons at the cellular level, we analyzed morphological and functional changes associated with intrinsic (astrocytes, microglia, and CNS-border macrophages) and extrinsic (infiltrating lymphocytes) cells, focusing on the telencephalon.

#### Astrocytes execute bi-directional control of lymphocyte fate at the perivascular-parenchymal interface in a spatiotemporal manner

**Fig 5:**
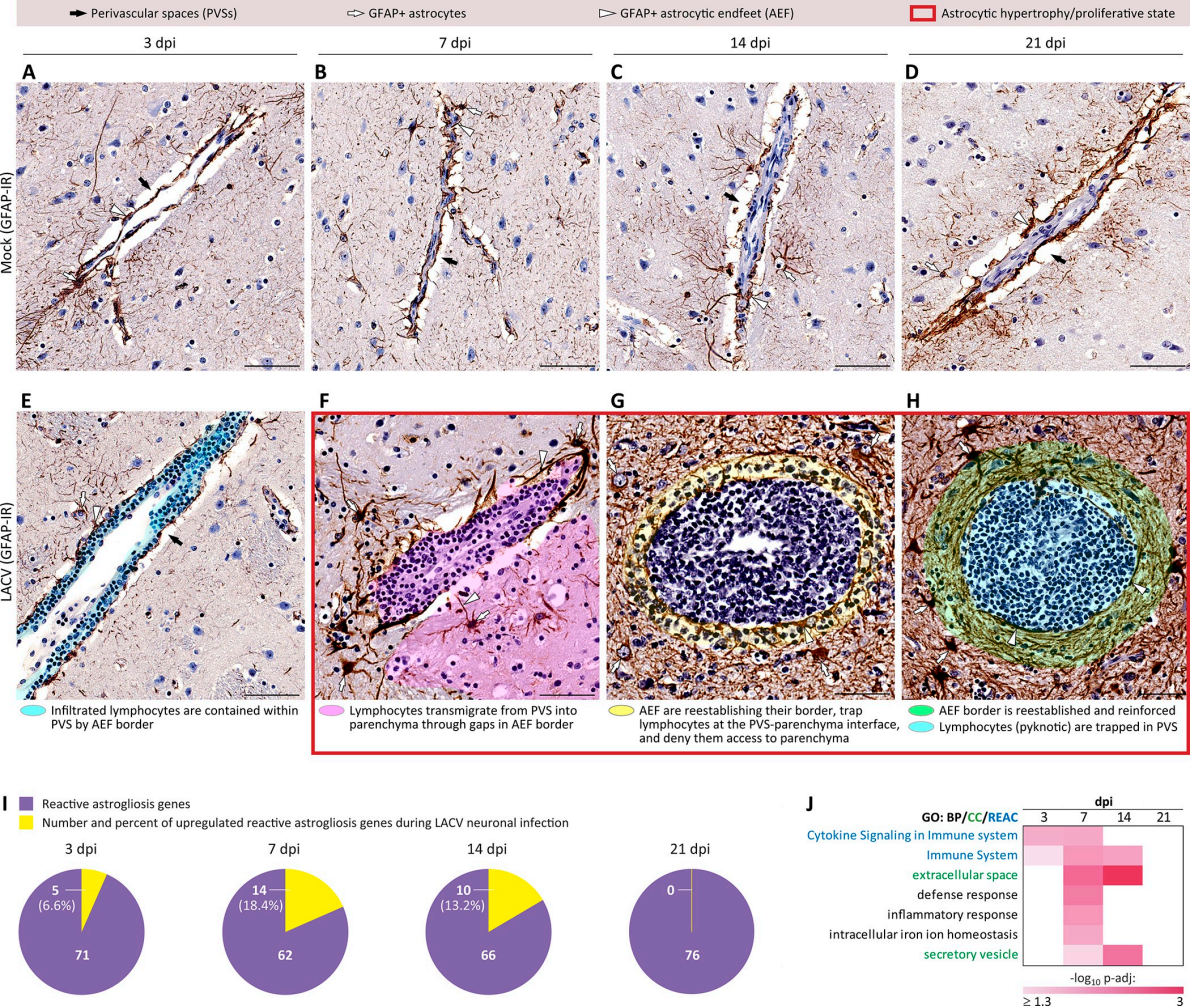
Time-limited bi-directional control of lymphocytic fate by reactive astrocytes at the perivascular-parenchymal interface during LACV infection of the CNS in NHPs. **(A—H)** Representative sections of the glial fibrillary acidic protein immunoreactivity (GFAP-IR; brown with blue counterstaining) show astrocytic behavior in the telencephalon at indicated dpi in mock (**A—D**) and LACV-infected NHPs (**E**—**H**). Labeling keys at the top apply to all panels in A—H. Labeling used to highlight the spatiotemporal changes in behavior of astrocytes and lymphocytes is provided below each corresponding panel in E—H. Note a reduced size and pyknotic appearance of lymphocytes within the territory overlayed in yellow at 14 dpi (G) and within the territory overlayed in cyan at 21 dpi (H). Supporting information for corresponding GFAP-IR in dpi matched LACV-infected NHPs other than shown in E—H is provided in S4I–S4L Fig. Scale bars (A—H): 50 μm. (**I**) Pie charts showing the overlaps (numbers and percentages) in genes that were upregulated during LACV infection and genes involved in reactive astrogliosis [34]. (**J**) Temporal heatmap (labeling as described in Fig 3) showing significant functional annotations based on all upregulated reactive astrogliosis genes identified in the overlaps (yellow in panel I).

First, we examined changes in morphology and spatiotemporal behavior of astrocytes during LACV infection using immunostaining for glial fibrillary acidic protein (GFAP) (Figs 5A–5H and S4I–S4L). At 3 dpi, astrocytes appeared to retain their basic morphology and non-proliferated state (Fig 5E; compare to mock in Fig 5A). The perivascular border formed by astrocytic endfeet (AEF) (glia limitans) appeared intact even though lymphocytes from the systemic circulation had already extravasated and infiltrated the perivascular spaces (PVSs). Subsequent time points revealed that astrocytes had acquired a reactive state with the key features [33] of hypertrophy (enlarged somata and thickened processes) and proliferation (hypercellularity with overlapping spatial domains) (Fig 5F–5H). At 7 dpi, astrocytes began to focally retract their AEFs, creating gaps in the perivascular AEF border through which lymphocytes began to transmigrate from the PVS into the adjacent parenchyma (Fig 5F). At 14 dpi, the AEFs began to extend back to the vascular wall, forming multiple enwrapping layers, trapping lymphocytes at the PVS-parenchyma interface, and preventing their further access to the parenchyma (Fig 5G). At 21 dpi, reestablishment of the perivascular AEF border was completed with a multilayer reinforcement, trapping the lymphocytes (which appeared pyknotic) within the PVS (Fig 5H).

To further dissect the reactive state of astrocytes at the molecular level, we identified overlaps between genes upregulated during LACV infection with known reactive astrogliosis signature genes [34]. We found that a relatively small percentage of genes upregulated during LACV infection belonged to the reactive astrogliosis signature, with the highest number at 7 dpi and none at 21 dpi (Fig 5I). Nevertheless, functional analysis of these overlapped genes indicated their role in the immune, defense, and inflammatory responses with a predominant location in the extracellular space (Fig 5J).

Taken together, this composite picture of spatiotemporal changes in astrocytic morphology, reactive state, behavior, and molecular expression suggests that the reactive astrocytes regulated lymphocytic influx into the CNS as an initial response to LACV infection, and later controlled lymphocytic fate by trapping the cells at the perivascular-parenchymal interface.

#### Lymphocytes undergo apoptosis in a spatiotemporal sequence starting from the parenchyma and progressing back to the original site of their extravasation into the perivascular space

**Fig 6:**
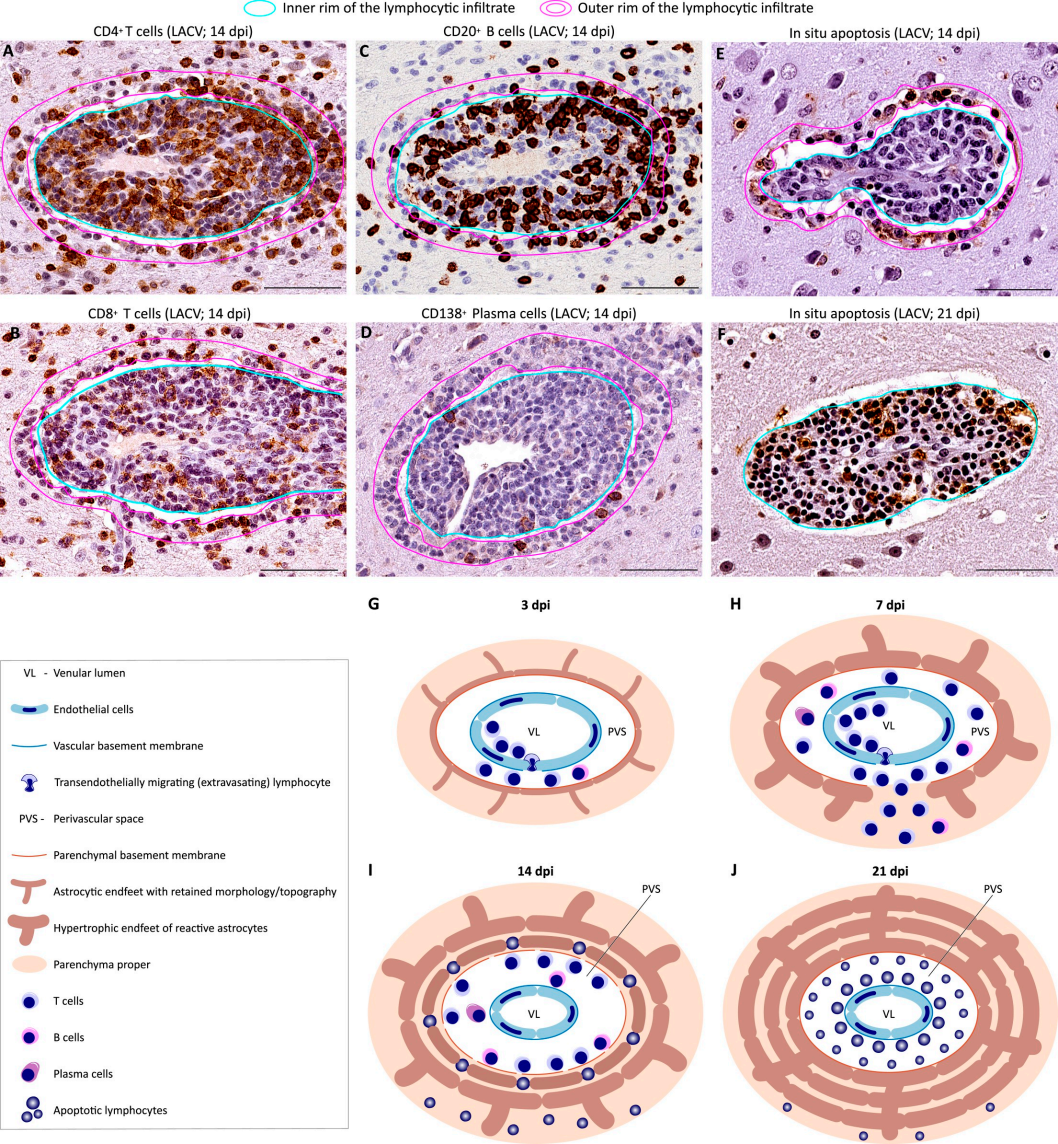
Phenotypic composition and fate of infiltrating lymphocytes during LACV infection of the CNS in NHPs. (**A—F**) Adjacent sections of the same venule in the telencephalon show the phenotype of lymphocytes at the peak of their infiltration (14 dpi) based on the immunoreactivity (brown with blue counterstaining) for indicated lymphocytic markers (**A**—**D**) and *in situ* apoptosis detection with ApopTag staining (brown) at 14 dpi (**E**) and 21 dpi (**F**). Labeling keys used in A—D are at the top of the figure. Scale bars (A—F): 50 μm. (**G**—**J**) Schematic summary of the fate of the infiltrating lymphocytes during neuronal virus infection that is spatiotemporally controlled by astrocytes (based on the findings shown in E, and F, and Fig 5E–5H. Anatomical landmarks and cell types are indicated in the box on the left. See text for a detailed description of the processes illustrated in G—J.

Next, we analyzed the phenotype of lymphocytes at their peak of infiltration into the CNS from systemic circulation (14 dpi) and their fate from 14 to 21 dpi of LACV infection. At 14 dpi, infiltrating lymphocytes were composed of the T cells (CD4+ and CD8+) (Fig 6A and 6B), B cells (CD20+) (Fig 6C), and antibody-producing plasma cells (CD138+) (Fig 6D). At that time, all types of lymphocytes were present in both PVSs and the adjacent parenchyma. However, a closer look revealed two separate topographical domains within perivascular lymphocytic infiltrates (outlined in cyan or magenta in Fig 6A–6D): (i) an inner rim adjacent to the venular wall (cyan) and (ii) an outer rim located in the immediately adjacent parenchyma (magenta). Remarkably, the outer rim was reminiscent of that seen during reestablishment of the AEF border by astrocytes at this time point (Fig 5G). Thus, the outer rim may represent the astrocytic trapping territory where lymphocytes are contained by AEF, denying them further access to the parenchyma.

Since the lymphocyte apoptotic process was transcriptionally upregulated at 14 dpi (Fig 3A) and lymphocytes had a reduced size and pyknotic appearance at 14 dpi (Fig 5G) and 21 dpi (Fig 5H), we investigated the fate of infiltrating lymphocytes at these time points by staining for apoptosis (see Materials and Methods). Lymphocyte apoptosis was first detected at 14 dpi and was localized to the outer rims of the perivascular lymphocytic infiltrates and in the adjacent parenchyma (Fig 6E). This indicates that the lymphocytes were undergoing apoptosis within the parenchyma and AEF trapping territory first (the latter is shown by yellow overlay in Fig 5G). However, at 21 dpi, the lymphocyte apoptosis signal had mostly disappeared from the parenchyma and AEF trapping territories, moving into the inner rims of perivascular infiltrates (Fig 6F). This confirms that lymphocytes of reduced size and pyknotic appearance (Fig 5G and 5H) were indeed apoptotic. These findings indicate that resolution of the lymphocyte-mediated inflammation is governed in time and space by astrocytes and lymphocyte apoptosis (schematically summarized in Fig 6G–6J).

In summary, we found that transcriptional upregulation and execution of lymphocyte apoptosis took place at 14 dpi, soon after the virus was cleared from neurons. From 14 to 21 dpi, apoptosis of lymphocytes started at the parenchyma and progressed back to the original site of their extravasation into the perivascular spaces.

#### CNS sequentially upregulates and downregulates the phagocytic cellular environment in response to virus infection

**Fig 7:**
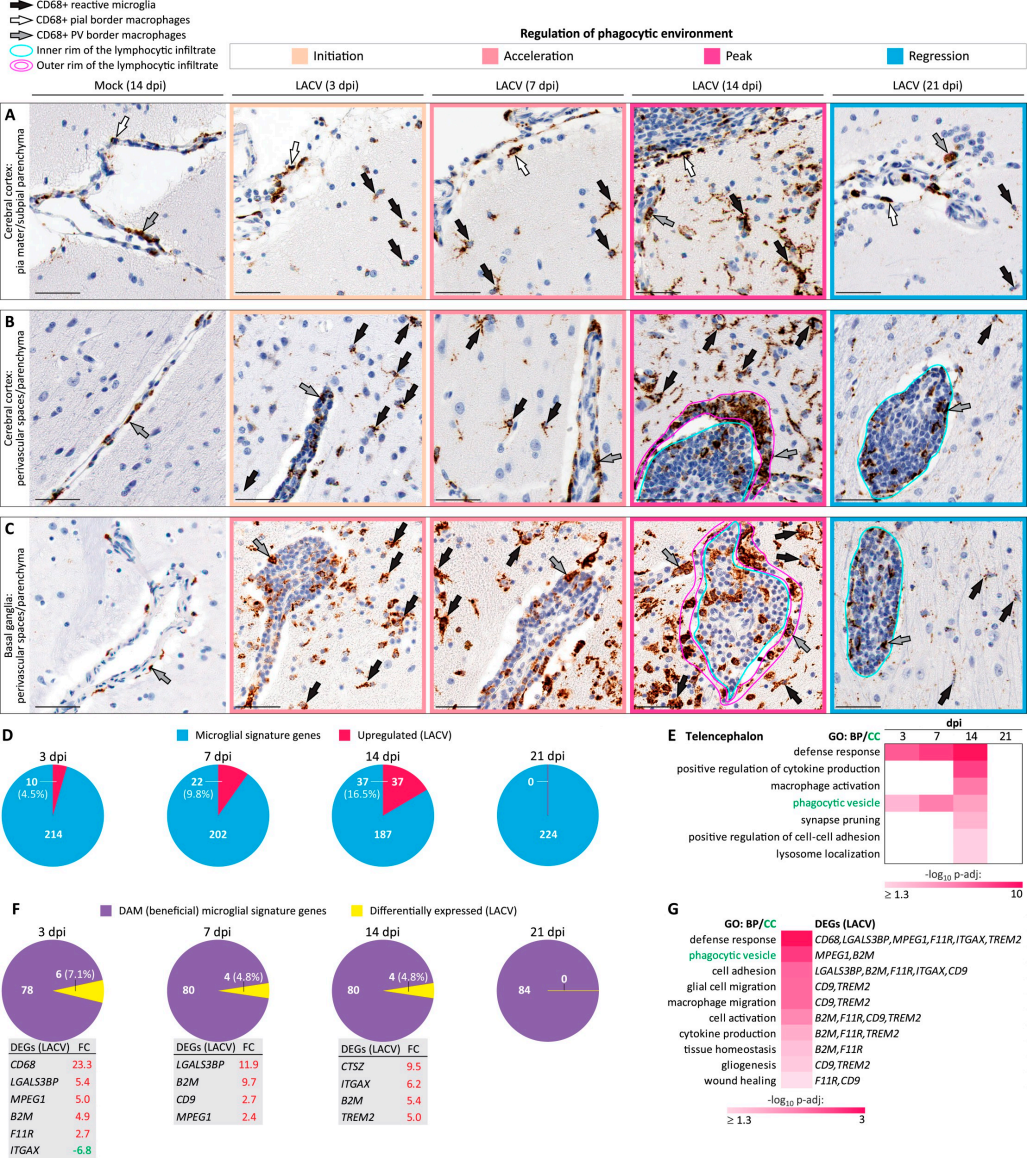
Regulation of phagocytic environment in the CNS of NHPs during LACV infection. **(A—C)** CD68-IR visualizes reactive microglia and CNS-border macrophages in the telencephalon. The cell labeling keys are referenced in the top left, followed by the color keys used to highlight the specific panels representing stages in regulation of the phagocytic environment. Each row compares representative morphological changes associated with CD68+ phagocytic cells indicated in the top left corner (brown with blue counterstaining) during LACV infection at indicated dpi (see S4M–S4P Fig for corresponding CD68-IR in dpi matched LACV-infected NHPs other than shown) and mock (14 dpi shown here as a reference for the peak changes in LACV; see S7 Fig for CD68-IR in the mock telencephalon at all time points). Scale bars (A—C): 50 μm. (**D—E**) Temporal dissection of functional microglial changes in the telencephalon based on the overlaps of genes differentially expressed during LACV infection and known sets of (i) microglial signature genes [35] (**D** and **E**) and (ii) disease-associated microglia (DAM) genes [36] (**F** and **G**). Overlapping differentially expressed genes (DEGs) with corresponding significant (p-adj < 0.05) log_2_ fold changes (FCs) are listed below the pie charts. (**E**) Temporal heatmap (labeling as described in Fig 3) shows significant functional annotations based on the upregulated microglial genes identified in the overlaps in panel D. (**G**) Significant functional genomic terms for all DEGs identified in the overlaps in panel F.

Finally, to investigate the roles of activated microglial cells and macrophages in resolution of inflammation, we examined the spatiotemporal behavior of these phagocytic cells using immunostaining for the phagocytic marker protein CD68. In the telencephalon of mock animals, CD68 immunoreactivity (CD68-IR) was detected in the CNS-border (pial and perivascular) macrophages at each analyzed time point (Fig 7 [first column] and S7 Fig). In the cerebellum of mock animals, CD68-IR was predominantly associated with the pial macrophages (S8A Fig), while the brainstem and spinal cord displayed rare CD68-positive satellite microglial cells abutting large neurons (S8B and S8C Fig). In contrast, in the LACV-infected telencephalon, the CNS-border macrophages and microglial cells underwent a time-limited change to a reactive phagocytic phenotype (i.e., increased CD68-IR, hypertrophy, foamy appearance, and engulfing morphology) (Fig 7A–7C). This change began at 3 dpi, accelerated at 7 dpi, peaked at 14 dpi, and regressed at 21 dpi. Similar kinetics of CD68-IR and morphological transformation of the pial macrophages and microglia was seen in the LACV-infected cerebellum (S8A Fig). Importantly, at 14 dpi, the same two rims (inner and outer) observed in the perivascular lymphocytic infiltrates with staining for apoptosis (Fig 6E) could also be clearly distinguished with CD68 staining (Fig 7B and 7C), with increased CD68-IR density and more macrophage-engulfed lymphocytes in the outer rim. By 21 dpi, the outer rim of CD68-IR had disappeared, and CD68+ macrophages were localized exclusively to the inner rim (perivascular space) (Fig 7B and 7C). These findings are consistent with the same spatiotemporal sequence of progression of the lymphocytic apoptosis (Fig 6E, 6F, 6I, and 6J) and indicate that the apoptotic lymphocytes were removed by phagocytosis.

The regression of the phagocytic environment at day 21 was associated with an overall reduction of CD68-IR in the pial, perivascular, and parenchymal locations and microglia returning to surveying homeostatic morphology with small somata and thin ramified processes (Figs 7A–7C and S8A). Development and regression of this phagocytic environment occurred only in the telencephalon (Fig 7A–7C) and cerebellum (S8A Fig), the CNS ROIs with virus-infected neurons (Fig 1F–1I and 1K) in which neurophysiological changes occurred during the first week of LACV infection (Figs 2 and S5). No such transformation in the state of the CNS-border macrophages and microglia was observed in the brainstem and spinal cord, the CNS ROIs with no detectable virus infection of neurons (Fig 1J, 1L and 1M) and with largely intact neurophysiology (S6 Fig). These results indicate that the development of the phagocytic environment in the CNS was a strictly proportional response to virus infection of neurons and neurophysiological changes.

To further dissect the reactive state of microglia at the molecular level, we identified overlaps between genes upregulated during LACV infection and known microglial signature genes [35]. We found a progressive increase in the number of upregulated microglial signature genes up to 14 dpi, but a complete cessation of this upregulation by 21 dpi (Fig 7D). Functional analysis of these overlapped genes indicated their role in the defense response and confirmed their role in the regulation of the phagocytic tissue environment (Fig 7E). Interestingly, nine microglial genes that were differentially regulated during the first two weeks of LACV infection (*CD68*, *LGALS3BP*, *MPEG1*, *B2M*, *F11R*, *ITGAX*, *CD9*, *CTSZ*, *TREM2*; Fig 7F) overlapped with the genes expressed by a unique beneficial phagocytic microglia (disease associated microglia [DAM] [36]) which has been reported to restrict the development of neurodegenerative diseases [37]. Functional analysis ascribed these overlapped genes to the defense response, phagocytosis, glial and macrophage cell migration, regulation of tissue homeostasis, and wound healing (Fig 7G).

Taken together, we found that in response to LACV neuronal infection, the CNS sequentially upregulates and downregulates the phagocytic cellular environment to execute tissue clearing that is time-limited and proportional to tissue damage.

### Integration of host responses to virus infection in the CNS of NHPs resulting in an optimal outcome

**Fig 8:**
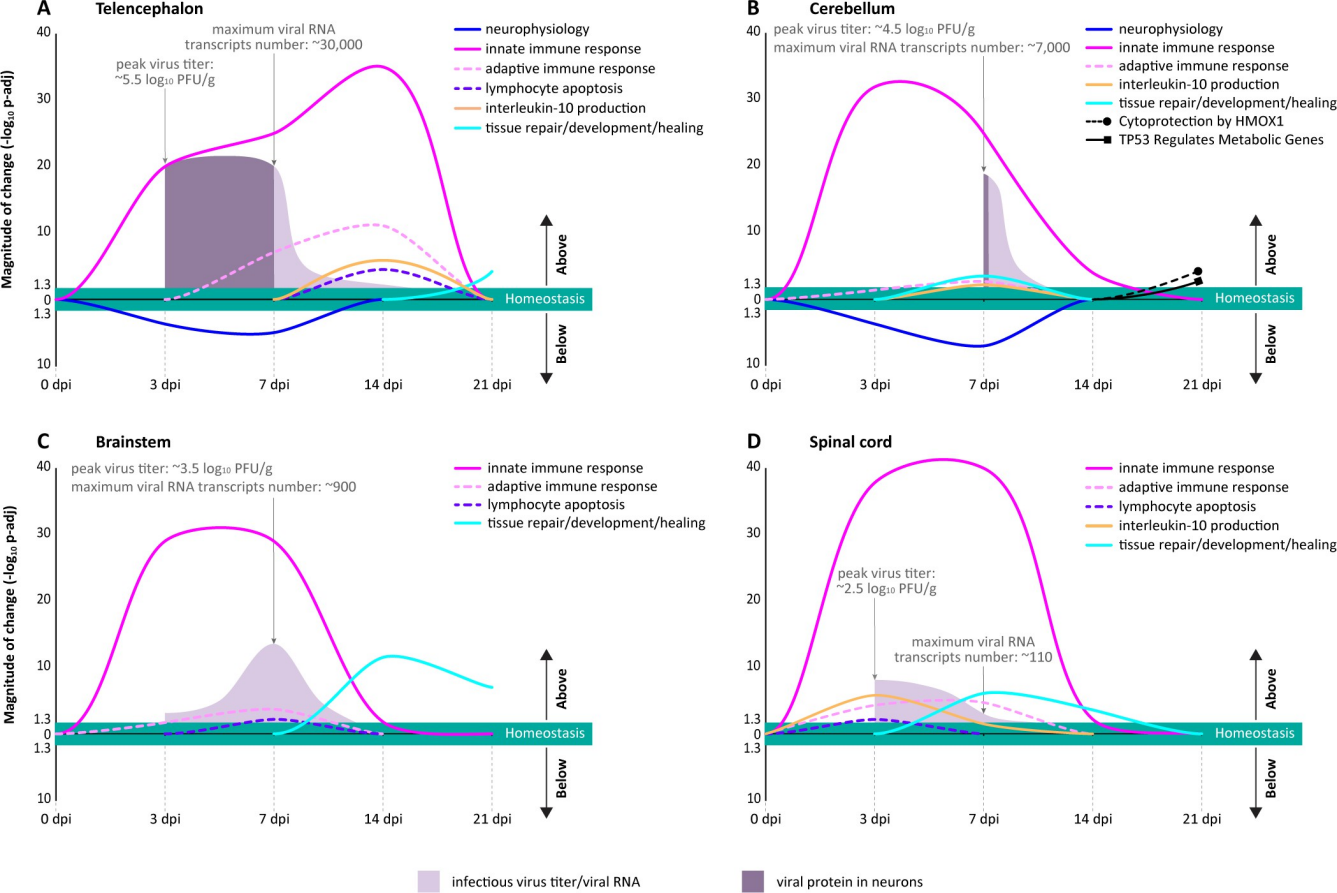
Spatiotemporal reconstruction of an optimal transcriptional regulation of host responses to virus infection of neurons in the CNS of NHPs. (**A—D**) Summary of major region-specific functional changes in the CNS transcriptome during 3 weeks after intrathalamic LACV inoculation. The plotted magnitude of change (y axes) for the selected biological processes and pathways in indicted CNS ROIs is based on the significance values (-log_10_ p-adj). The territory of the homeostasis (green area in each plot) is statistically defined as being not significantly different from the normal physiological conditions (i.e., dpi-matched mock) at a cut-off of 1.3 -log_10_ p-adj (FDR-adjusted p < 0.05). The indicated functional terms were manually collapsed based on the enrichment data presented in Figs 2A, S5A, S6A, 3, and 4. For the areas above the homeostasis territory (upward arrows in each graph), the upward direction in the plotted curves represents active transcriptional upregulation, whereas the downward direction implies cessation of transcriptional upregulation, active transcriptional downregulation, or both. For the areas below the homeostasis territory (downward arrows in each graph), the downward direction in the plotted curves (present only in A and B) represents active transcriptional downregulation, whereas the upward direction implies cessation of transcriptional downregulation, active transcriptional upregulation, or both. The shaded areas (keys are at the bottom of the figure) are derived from Fig 1 and superimposed to show the kinetics of virus replication (peak titers of infectious virus and maximal numbers of viral RNA transcripts) and production of viral protein in neurons.

NHPs infected intrathalamically with LACV did not display any overt signs of neurological impairment. While the virus infected and replicated in the neurons during the first week of infection, the CNS cleared the infectious virus, viral protein, and viral RNA rapidly within three weeks after onset (Fig 8). Furthermore, virus replication and the accompanying host responses during the first week of infection had only a mild and transient impact on the neurophysiology that was fully reversible. To integrate and better understand how the CNS of primates optimally controlled LACV infection of neurons and mitigated neurophysiological damage, we reconstructed the transcriptional and cellular responses in the CNS, focusing on three major functional categories: neurophysiology, immune response (innate and adaptive), and tissue repair.

The CNS of NHPs responded to LACV infection with a transient transcriptional downregulation of neurophysiological functions limited to the telencephalon and cerebellum (Fig 8A and 8B), the sites of the CNS in which the virus infected neurons. The peak in these transcriptional changes occurred at 7 dpi, strictly coinciding with the timing of virus protein detection in neurons (Fig 1F–1I and 1K). Importantly, this transcriptional downregulation returned to normal homeostasis by 14 dpi. This indicates that the neurophysiological processes in the CNS during virus infection are tightly transcriptionally regulated in space and time and strictly proportional to the degree of damage inflicted on neurons by viral replication.

Transcriptional upregulation of innate immune responses in CNS of NHPs infected with LACV was rapid and occurred in all CNS regions examined, regardless of whether detectable virus was present in the neurons (Fig 8A–8D). However, the subsequent kinetics of innate immune responses was dependent on the infectious status of neurons. There was a faster and more precipitous decline and return to homeostasis in the uninfected brainstem and spinal cord (Fig 8C and 8D), compared to the infected telencephalon and cerebellum (Fig 8A and 8B). The same general kinetics were observed for transcriptional regulation of adaptive immune responses. However, transcriptional upregulation of adaptive immune responses in the telencephalon was higher in magnitude and lasted longer (Fig 8A), compared to all other CNS regions (Fig 8B–8D). The peak of this upregulation coincided with the peak in upregulation of lymphocyte apoptosis. Importantly, transcriptional regulation of both innate and adaptive immunity, as well as processes leading to the resolution of inflammation (e.g., lymphocyte apoptosis and production of IL-10, an anti-inflammatory cytokine), returned to homeostasis between 14 and 21 dpi.

Transcriptional initiation of tissue repair, development, and healing processes in the telencephalon coincided with restoration of normal neurophysiological regulation (at 14 dpi). Notably, these reparative processes became transcriptionally activated in the cerebellum, brainstem, and spinal cord a week earlier (3–7 dpi) than in the telencephalon. This suggests that the delay in activation of the reparative processes in the telencephalon was needed to ensure that the virus infection was completely cleared from neurons and inflammation could be resolved safely. In addition, transcriptional activation of tissue repair included two noteworthy biological processes: anti-inflammatory (Cytoprotection by HMOX1) and homeostatic (*TP53* Regulated Metabolic Genes) activities (Fig 8B).

Next, we analyzed the events in the CNS during LACV infection that were downstream of changes in gene expression by integrating spatiotemporal changes in the cellular protein expression, morphology, topology, and functions of neurons, astrocytes, lymphocytes, microglia, and CNS-border macrophages (Table 1). The integrated multicellular response to virus infection of neurons in the CNS of NHPs involved the following events: i) the structural integrity of neurons was transiently disrupted in the telencephalon and cerebellum, peaking at 7 dpi, subsiding at 14 dpi, and completely recovering by 21 dpi; (ii) reactive astrocytes orchestrated a bi-directional control of the migration and fate of infiltrated lymphocytes at the perivascular-parenchymal interface; (iii) at 14 dpi and afterwards, infiltrated lymphocytes underwent programmed cell death; and (iv) microglia and CNS-border macrophages phagocytosed apoptotic lymphocytes and created a milieu for tissue repair.

**Table 1:** Integration of functional cellular changes in the CNS during an optimal handling of neuronal virus infection.

Cell type	Spatiotemporal changes in the cellular protein expression/morphology/topology/functions				Reference figures
	3 dpi	7 dpi	14 dpi	21 dpi	
**Neurons**	• LACV + (BG) • MAP2-IR ↓ and dendritic beading (BG) • axonal torpedoes • TH-IR ↓ (BG)	• LACV + (BG, CX, CB) • MAP2-IR nonuniform (CX, CB) • MAP2-IR ↓ (BG) • PanN-IR ↓/axonal torpedoes (BG) • TH-IR ↓ (BG), ↓ (SN)	• MAP2-IR nonuniform (CX) • MAP2-IR ↓ (BG) • MAP2-IR focal depletion (CB) • PanN-IR ↓ (BG) • TH-IR ↓ (BG)		Fig 1E–1N, Fig 2B–2F, S3 Fig, S4A–S4H Fig, S5B –S5I Fig
**Astrocytes**		• GFAP ↑ (TE) • hypertrophy of somata/ processes/AEF • AEF retraction forming gaps in AEF border	• GFAP ↑ (TE) • hypertrophy of somata/ processes/AEF • hypercellularity/proliferative state • reestablishment of AEF border • AEF entrap apoptotic lymphocytes within outer rim of PV infiltrates	• GFAP ↑ (TE) • hypertrophy of somata/processes/AEF • hypercellularity/proliferative state • AEF border is reestablished and reinforced by multiple concentric layers of astrocytic processes/AEF	Fig 5E–5H, Fig 6G–6J, S4I–S4L Fig
**Lymphocytes**	• extravasation into PVSs of venules (BG)	• PV infiltration/transmigration into parenchyma (BG, CX) • Pial infiltration (TE, CB)	• Phenotypic composition (TE): • T cells (CD4+ and CD8+) • B cells (CD20+ and CD138+) • Lymphocyte apoptosis within outer rim of PV infiltrates	• Lymphocyte apoptosis within inner rim of PV infiltrates (TE)	Fig 5E–5H, Fig 6, Fig 7B and 7C, S4I–S4P Fig, S5G–S5I Fig
**Microglia/** **CNS border macrophages**	• CD68-IR ↑ (TE, CB) • Initiation of PV, pial, sub-PV-parenchymal phagocytosis	• CD68-IR ↑ (TE, CB) • Acceleration of PV, pial, sub-PV-parenchymal phagocytosis	• CD68-IR ↑ (TE, CB) • Peak of PV, pial, sub-PV-parenchymal phagocytosis	• CD68-IR ↓ (TE, CB) • Regression of PV, pial, sub-PV-parenchymal phagocytosis	Fig 7A–7C, S8A Fig

↓ decreased compared to dpi-matched mock. ↑ increased compared to dpi-matched mock. IR, immunoreactivity. MAP2, microtubule associated protein 2; PanN, pan-neuronal antibodies; TH, tyrosine hydroxylase; GFAP, glial fibrillary acidic protein. BG, basal ganglia. CX, cerebral cortex. TE, telencephalon (BG and CX). CB, cerebellum. SN, substantia nigra. AEF, astrocytic endfeet. PV, perivascular. PVSs, perivascular spaces.

Inferred functional change: MAP2-IR ↓/nonuniform/focal depletion—disruption of structural integrity of the neuronal somatodendritic compartments. PanN-IR ↓/axonal torpedoes—disruption of structural integrity of the neuronal somata, dendrites, and axons. TH-IR ↓—disruption of structural integrity and neurotransmission in the dopaminergic neurons (SN) and their projections (BG). GFAP-IR ↑—reactive astrogliosis with hypertrophy of somata/ processes/AEFs, hypercellularity, and proliferative state. CD4+, helper T cell infiltration. CD8+, cytotoxic T cell infiltration. CD20+, B cell infiltration. CD138+, plasma (antibody producing) cell infiltration. CD68-IR ↑—establishment of phagocytic environment by microglia/CNS-border macrophages.

Collectively, these findings provide the spatiotemporal profile of a well-coordinated transcriptional and cellular regulation of the primate CNS defense response to viral infection of neurons. This regulation should be considered as optimal since it encompassed complete virus clearance, rapid activation and timely termination of immune responses, resolution of inflammation, and initiation of tissue repair, while being strictly proportional in space and time.

## Discussion

In this study we show that the CNS of immunocompetent NHPs can mount effective molecular and cellular responses to a virus infection of neurons, successfully minimize neurophysiological disturbances, and restore homeostasis. Our current understanding of how the human CNS successfully controls virus infections of neurons and recovers over time is very limited. In humans, CNS biopsies are rarely performed and typically available at only one time point during life, or tissue is obtained postmortem when the patient has succumbed to infection. Therefore, we studied virus CNS infection in rhesus monkeys which are evolutionary, physiologically, and immunologically similar to humans [38]. Animals were inoculated intracerebrally with LACV that can cause encephalitis in humans, but it is rarely fatal [15,16]. Intracerebral delivery of the virus was the only feasible route to consistently establish LACV infection in the CNS, since peripheral inoculation of immunocompetent NHPs that would mimic the natural route of LACV infection by a mosquito bite elicits high titers of virus neutralizing antibodies in the blood, that might prevent virus invasion of the CNS [17]. The same phenomenon has necessitated the use of intracerebral inoculation of virus to model the CNS infection in NHPs with West Nile virus (WNV) [39]. This is consistent with the epidemiology of WNV infection in humans, as fewer than 1% of all infected people develop infection of the CNS [40]. While most LACV infections in humans are thought to be asymptomatic [16,41], the actual percentage of infected individuals who develop the LACV neurological disease (LACV-ND) is unknown. Therefore, peripheral inoculation of immunocompetent NHPs with LACV to study infection of the CNS is unrealistic since it would require a very large number of animals to induce disease in only a few. On the other hand, the intracerebral NHP model satisfied several requirements to study the optimal response to virus infection in the CNS. First, age-appropriate juvenile rhesus monkeys (2–3 years old) were used, which approximated the ages of young children at most risk of LACV-ND [41,42] (~7–9 years old, based on an estimated 3.2-fold age differential between rhesus monkeys and humans [43]). Second, intracerebral inoculation of LACV resulted in infection of neurons and recapitulated the predominant neuropathology in the cerebral cortex and basal ganglia (telencephalon), the less pronounced involvement of the cerebellum, and the sparing of the brainstem and spinal cord in children with LACV-ND [23]. This differential anatomical involvement in children presents as seizures, altered mental statis, headaches, upper extremity paresis with a positive Babinski sign, and choreoathetosis (due to involvement of the telencephalon), while ataxia (due to involvement of the cerebellum) is less common [15,42,44,45]. Sparing of the spinal cord correlates with the absence of acute flaccid paralysis in patients with LACV-ND [46]. Third, despite LACV replication in neurons during the first week after inoculation, the impact on neurophysiological processes was transient and did not produce overt neurological signs in NHPs, indicating that they successfully controlled the infection and recovered.

Our findings reveal that the favorable outcome of the CNS infection after LACV inoculation in NHPs was achieved by effective transcriptional regulation of antiviral host defense responses and downstream reactive changes in CNS intrinsic and extrinsic cells. We view these molecular and cellular responses as optimal since they (i) led to rapid viral clearance from infected CNS regions, (ii) were restricted in time and space and strictly proportional to virus replication and neuronal damage, (iii) were resolved after virus clearance in a timely manner, (iv) restored neurophysiological homeostasis, and (v) initiated tissue repair. This contrasts with infection of the primate CNS with WNV, in which virus replication and transsynaptic spread between connected neurons that govern control of movement [13], coupled with rising virus loads and exuberant host defense responses, lead to severe neurological impairment as early as 9 dpi [14,39]. In this study, LACV infected only upper motor neurons (i.e., corticospinal motor neurons [47], known as Betz cells in humans [48]) in the parietal (primary motor) cortex during the first week. Remarkably, in contrast to the anterograde axonal spread of WNV from upper to lower motor neurons [13], lower motor neurons in the spinal cord of NHPs [49] infected with LACV remained intact. This suggests that the potential for spread of LACV along the corticospinal tract [50,51] was blocked. The use of NHPs in this study is important for modeling human infection since the corticospinal motor system in humans and NHPs are similar, and differ in rodents [52].

We found that the transcriptional program used by the primate CNS to successfully control virus infection without irreversible damage to neural functions is strictly proportional to the infectious status of neurons and involves active downregulation of host defense responses soon after the virus is cleared from neurons. Resolution of inflammation in the CNS parenchyma is a highly regulated and active process that should be completed before tissue homeostasis can be restored [53]. This process relies on the coordination of functions of the CNS-resident cells, as well as cells recruited from the systemic circulation [54]. Our findings are consistent with the current view regarding the sequence of events that should take place to resolve inflammation in the CNS after injury [54,55]. On the other hand, dysregulation of this process may result in chronic inflammation and further tissue injury [56,57]. In this study, the optimal regulation of host responses to virus infection that led to resolution of inflammation in the CNS was associated with the following downstream coordinated activities of CNS intrinsic and extrinsic cells (Table 1): (i) bi-directional control of lymphocyte infiltration by astrocytes through first facilitating their access to the infected CNS parenchyma and later by trapping them at the perivascular-parenchymal interface, (ii) termination of the adaptive immune response in the CNS by apoptosis of the lymphocytes, and (iii) elimination of apoptotic lymphocytes by reactive microglia and CNS-border macrophages. It is likely that any change in regulation of these orderly processes may result in an unfavorable outcome for the host with inability of the CNS to clear virus from neurons and/or failure to control structural and functional damage, resolve inflammation, restore homeostasis, and repair the tissue. Deviations from the optimal regulation of host responses to viral infection in the CNS may arise in patients with primary or secondary immunodeficiencies [58,59], including those with loss or gain of protein function due to monogenic inborn errors of immunity [60,61]. Inborn errors of immunity can predispose to viral encephalitides caused by relatively benign viruses, especially in children, by disruption of cellular immunity in the CNS, which can result in severe infections of specific CNS regions such as the forebrain [62] or brainstem [63]. While children with LACV-ND are reported as having no known immunocompromising condition [42], this does not rule out an undiagnosed inborn error of immunity. Increased availability of whole genome sequencing may facilitate a genetic diagnosis in some patients with viral CNS infections and provide a better understanding of the pathogenesis of infection and improve outcomes.

A remarkable feature of the optimal CNS response to LACV infection in this study was the highly coordinated sequence of events orchestrated by astrocytes, lymphocytes, microglia, and CNS-border macrophages. As an efferent arm of the adaptive immune response to virus infection in the CNS [4,64], lymphocytes are actively recruited from the systemic circulation and cross several barriers at the vascular-parenchymal interface [65]. Lymphocyte entry into the CNS parenchyma is controlled by astrocytic endfeet that form a continuous perivascular border (glia limitans) and restrict infiltration of cells from the blood [66]. In the LACV-infected CNS, we found that lymphocyte transmigration from the blood and subsequent infiltration into the adjacent parenchyma were highly regulated in space and time by astrocytes. This regulation was bi-directional, as astrocytes first retracted their endfeet to allow lymphocytes to progress into the parenchyma and then, after about one week, extended their hypertrophic endfeet back and closed the perivascular border by re-establishing perivascular astrocytic endfeet coverage. This was followed by reinforcement of the perivascular border by forming multiple layers of hypertrophic astrocytic endfeet that tightly enwrapped perivascular lymphocytic infiltrates. This suggests that by trapping the infiltrated lymphocytes at the perivascular-parenchymal interface after LACV was cleared from neurons, the astrocytes controlled the function and fate of lymphocytes (including T and B cells, as well as antibody-producing plasma cells), ultimately resulting in their elimination. Control of trafficking of adaptive immune cells from the circulation to the parenchyma by astrocytes may be a common mechanism employed by the CNS during inflammation due to infection, trauma, or autoimmunity [66,67].

We also found that lymphocyte apoptosis, one of the important mechanisms to resolve inflammation and restore tissue homeostasis in the CNS [53,68], was first initiated in the parenchyma and at the perivascular-parenchymal interface where the lymphocytes were trapped by astrocytic endfeet and that lymphocyte apoptosis progressed to perivascular spaces, the sites of initial lymphocyte extravasation. This suggests that astrocytes, which are capable of abrogating lymphocytic responses by inducing apoptosis [69], controlled the spatiotemporal progression of lymphocyte apoptosis in the CNS of LACV-infected NHPs. Apoptosis of perivascular lymphocytes, in an effort to control inflammation, has been reported in the CNS of NHPs during other viral infections; both transcriptional upregulation of lymphocyte apoptosis [14] and apoptotic lymphocytes in perivascular infiltrates [12] are prominent features of CNS infections with neuropathogenic flaviviruses. The coordinated spatiotemporal sequence of events during interactions between astrocytes and infiltrating lymphocytes in the CNS of NHPs offers a better understanding of the resolution of inflammation in the CNS of humans, where it is impossible to visualize and study these events during disease progression [70]. In addition, human astrocytes are unique in many morphological and functional aspects, including structural features, increased regional diversity, specialized transcriptomic profile, and diversity of functions [71]. These features of human astrocytes should be carefully considered when interpreting experimental data from mice and attempting their extrapolation to humans. For example, compared to mice, human astrocytes have higher expression of genes regulating defense responses, processes in the extracellular space, and secreted cytokines [72]. Remarkably, we found that these exact processes were transcriptionally regulated by astrocytes during LACV infection in NHPs, adding more weight to the translational potential of this primate model. These findings are important since the roles of astrocytes in viral infections of the CNS are only beginning to be elucidated and are an active area of research [73].

Our study has some potential limitations. As noted above, we inoculated NHPs with LACV directly into the brain parenchyma, rather than infecting the animals peripherally to mimic natural infection by a mosquito bite. The limitations and ethics of using a large number of NHPs to obtain CNS infection in a few precluded the use of peripheral inoculation. In addition, although our findings demonstrate transcriptional and translational features of efficient virus clearance, resolution of inflammation, and restoration of CNS homeostasis after LACV infection, NHPs in this study were evaluated at various time points up to 3 weeks. Future studies beyond 3 weeks may offer additional insights into functional changes associated with CNS-intrinsic and extrinsic cells over time.

Finally, we found that resolution of inflammation and establishment of the milieu for tissue repair in the CNS of LACV-infected NHPs was facilitated by reactive microglia and CNS-border macrophages (i.e., CNS-intrinsic phagocytic immune cells [74]). Strikingly, phagocytosis executed by these cells followed the same spatiotemporal sequence as lymphocyte apoptosis (initiating in the parenchyma and progressing to the perivascular spaces). We note that the repertoire of the microglial transcriptome in the telencephalon during LACV infection has many similarities with a recently described microglial phenotype [36] thought to restrict development of neurodegeneration [37]. This suggests that the microglial transcriptome made an important contribution to the optimal host response to LACV infection in the CNS. In addition, we identified transcriptional upregulation of two important anti-inflammatory and homeostatic pathways that coincided with resolution of inflammation and initiation of tissue repair in the CNS. These pathways are related to cytoprotection by regulation of heme oxygenase-1 [28–31] and regulation of homeostasis by activation of the *TP53* gene [32]. Thus, these findings have important implications for therapeutic targets to improve outcomes of viral CNS infections.

## Materials and methods

### Ethics statement

All animal procedures were performed according to Animal Welfare Regulations (USDA), Public Health Service Policy on Humane Care and Use of Laboratory Animals, the Guide for the Care and Use of Laboratory Animals and the animal study proposal (#LID 5E) was approved by the Institutional Animal Care and Use Committee (National Institutes of Health, Bethesda, MD).

### Study design

The study was conducted in twelve juvenile (~2–3 years old) rhesus monkeys (*Macaca mulatta*), obtained from the NIAID Morgan Island Breeding Program, Yemassee, SC. The monkeys were screened for neutralizing antibodies against LACV and were confirmed seronegative. The virus used was LACV/78/WI-H isolated from postmortem brain tissue of a patient who died after developing encephalitis in Wisconsin in 1978. The virus underwent several passages (one passage in mouse brain, two passages in BHK-21 cells, and one passage in Vero cells) and was biologically cloned by terminal dilution in Vero cells [17]. Eight monkeys were inoculated intrathalamically bilaterally with a total dose of 5 log_10_ plaque forming units (PFU) of LACV (0.2 ml inoculum volume for each inoculation site) and four monkeys were mock-inoculated in the identical manner with the same volume of Leibovitz’s L-15 medium (Invitrogen; Carlsbad, CA). The optimized procedure of bilateral intrathalamic inoculation of NHPs is described in detail elsewhere [75]. Briefly, each anesthetized monkey’s scalp was shaved and disinfected and the animal was placed in a recumbent position with the ventral side down. The parietal and temporal sutures of the skull were located and marked. The anatomical coordinates of two inoculation sites were 5 mm posterior to the temporal sutures and 15 mm equidistant from the parietal suture. After making two small incisions (each approximately 5 mm long in the coronal plane) over identified inoculation sites through the skin and galea aponeurotica, two holes (0.8 mm in diameter) were drilled through the skull and the inoculum (0.2 ml volume) was injected into the left and right thalamic regions at a low and steady rate. After both injections were completed, the scalp incisions were closed with Vetbond (3 M, St. Paul, MN).

Animals were observed twice daily for overt neurological signs such as incoordination, limb weakness, tremors, and seizures according to previously developed guidelines [75].

Serum samples to determine viremia and titers of LACV-neutralizing antibodies were collected on day 0, 3, 5, 7, 10, 14, and 21 after the intrathalamic inoculation procedure from all LACV-infected and mock animals that remained available at each specific time point of the study. Cerebrospinal fluid (CSF) samples were collected from each animal immediately before the necropsy.

Necropsies were performed on euthanized animals after cardiac perfusion with sterile saline at 3, 7, 14, and 21 days postinoculation (dpi) with all possible precautions taken to prevent virus cross-contamination between animals and between samples from the same animal, as well as to minimize technical inter-animal variability. These included: (i) performing aseptic procedures using sterile disposable instruments for each animal during removal of the brain and spinal cord; (ii) on the same day, performing aseptic necropsy on the mock animals first, followed by the aseptic necropsy LACV-infected animals; (iii) performing aseptic procedures using sterile disposable instruments for each coronal/sagittal/transverse cut during neuroanatomical tissue dissection, with uniform sampling of all major CNS regions in all animals (described in more detail below); (iv) immediately preserving all samples in individual sterile containers according to the requirements for specific downstream analyses used in this study (i.e., fresh frozen samples for virology and formalin fixed paraffin embedded [FFPE] tissues for correlative molecular pathology and RNA-seq). A graphic abstract of the study design is given in Fig 1A and 1B.

### Neuroanatomical tissue dissection

Brain and spinal cord were aseptically removed in their entirety from each animal and immediately aseptically dissected to include all major CNS regions based on the neuroanatomical coordinates in millimeters anteriorly and posteriorly from the anterior commissure (“ac”) [27] and similar to a previous description (supplemental Fig 1 in [75]). Briefly, the brain was cut parasagittally (approximately 3 mm to the left from the midline) to avoid tissue distortion during formalin fixation by preserving the midline structures.

After the parasagittal cut, the left hemisphere of the brain (Fig 1A) was aseptically coronally dissected using the “ac” coordinates as above and samples collected into separate sterile containers, immediately frozen and stored at -80°C until virus titration in Vero cells. The contralateral brain hemisphere was immediately immersed in 10% formalin and fixed for 7 days. The formalin fixed right hemisphere (with a portion of a parasagittal midline structures) was dissected coronally in the manner identical to the left hemisphere, except the cerebellum and brainstem were dissected sagittaly to maximize tissue representation in each tissue section for downstream molecular pathology and RNA-seq analyses (see Fig 1A and 1B and S1 Fig for more detail). The CNS regions analyzed were the cerebral cortex (frontal, temporal, parietal, and occipital), basal ganglia, thalamus, brainstem, cerebellum, and spinal cord.

### Detection of infectious virus

Virus titers in the serum, CSF, and CNS tissue homogenates from all CNS ROIs (Fig 1A) were determined by the plaque-forming assay as previously described [25].

### Detection of LACV-infected cells in the CNS

Detection of LACV-infected cells in all CNS ROIs was performed by immunohistochemistry on 5 μm FFPE sections using mouse monoclonal antibody (hybridoma CRL-2287; ATCC; 1:1000) against the Gc glycoprotein of LACV. Antigen retrieval was performed by boiling sections in Diva Decloaker (Biocare Medical). Further processing for colorimetric detection was according to the instructions for the MACH 4 universal polymer detection kit (Biocare Medical), and diaminobenzidine (DAB; brown) was used as a chromogen. Sections were counterstained with hematoxylin (blue). Positive controls included sections prepared from pelleted Vero cells infected with LACV. Negative controls included omission of the primary antibody step, as well as FFPE sections prepared from uninfected pelleted Vero cells and uninfected CNS tissue of rhesus monkeys.

### Detection of virus neutralizing antibodies

Detection of the neutralizing antibodies against LACV/78/WI-H virus in the serum and CSF samples was carried out by a plaque reduction neutralization assay and 60% plaque-reduction neutralization titers were determined as previously described [17]. The LACV neutralizing antibody titer was defined as the dilution of a sample that neutralized 60% of the virus.

### RNA-seq

Total RNA was isolated from freshly cut FFPE tissue sections that contained the selected CNS ROIs (see Digital pathology methods below) using the miRNAeasy FFPE kit, following the manufacturer’s protocol with minor modifications (Qiagen, Valencia, CA). Briefly, for each tissue block, 5 μm FFPE sections were placed in a 1.5 mL tube and deparaffinized with 160 μL Deparaffinization Solution (Qiagen). Decrosslinking was performed for 1 hour at 80°C. Purified total RNAs were quantified with the Qubit 4 fluorometer and the RNA High Sensitivity assay (Invitrogen, Waltham, MA) and assessed for degradation using the 2100 Bioanalyzer and the RNA 6000 Pico chip assay (Agilent Technologies, Valencia, CA). The DV100 and DV200 values representing the percentages of RNA fragments above 100 and 200 nucleotides in length, respectively, were determined from 2100 Bioanalyzer software.

Ribosomal RNAs were depleted from 100 ng total FFPE RNA using the QIAseq FastSelect -rRNA MHR kit and following the FastSelect protocol for KAPA RNA HyperPrep kit, with no fragmentation (Qiagen, Valencia, CA). Sequencing libraries were generated using the Kapa RNA HyperPrep library preparation and technical data sheet KR1350 v3.20, with minor modifications (Roche Sequencing Solutions, Indianapolis, IN). The Kapa Universal Adapter and UDI primers were utilized to create indexed libraries with 21 PCR amplification cycles. To obtain sequence reads per μl for creating a balanced library pool, a 1 μl aliquot of each library was combined into a single pool, quantified using the Kapa Library Quantification Kit (Roche Sequencing Solutions, Indianapolis, IN), and sequenced as 2 X 150 bp paired end reads on the MiSeq instrument using the Miseq Reagent Nano, v2, 300 cycle kit (Illumina, San Diego, CA). A balanced library pool was generated using the data from the Miseq run, quantified as before, and sequenced as 2 X 100 bp paired end reads on the Novaseq 6000 using the Novaseq S2, 200 cycle kit.

The amount of LACV RNA was measured in FFPE tissues from all CNS ROIs (Fig 1A and 1B) of LACV-infected and mock NHPs by RNA-seq based on the number of LACV sequence reads aligned to the sequence of inoculated LACV (https://www.ncbi.nlm.nih.gov/nuccore/EF485035) per 100 ng of RNA.

Gene expression data files from this study have been deposited to the GEO repository with accession number GSE266209 (https://www.ncbi.nlm.nih.gov/geo/query/acc.cgi?acc=GSE266209).

### Analysis of differential gene expression

Since the clustering analysis (S9 Fig) consistently showed a high similarity in gene expression between the biological replicates (i.e., NHP #1 and NHP #2) in all CNS ROIs at specific dpi, the gene expression data for these replicates was averaged. Bioconductor package DESeq2 [76] was used to identify significantly differentially expressed genes (DEGs) (Benjamini-Hochberg false discovery rate [FDR] adjusted p values [p adj] < 0.05; log_2_ fold change ≤ -2 and ≥ 2) between the following biological conditions: (i) “disease time points versus normal”—comparing the CNS ROIs (Figs 1B and S1) at each time point (3, 7, 14, and 21 dpi) versus dpi-matched mock; and (ii) “progression of disease over time”– comparing the CNS ROIs of a given time point to a preceding one (i.e., 7 vs. 3 dpi; 14 vs. 7 dpi, and 21 vs. 14 dpi).

### Functional genomic analysis

Functional enrichment analyses of the differential gene expression data were performed using the multi-source gProfiler platform [77] to identify significantly enriched gene ontology (GO) terms and Reactome/KEGG pathways. The lists of significantly upregulated or downregulated genes in each CNS ROIs (i.e., telencephalon, cerebellum, brainstem, and spinal cord) for the biological conditions described above were inputted simultaneously into gProfiler and run as 4-way multiquery. g:SCS threshold or Benjamini-Hochberg FDR (https://biit.cs.ut.ee/gprofiler/page/docs#significance_threhshold) was used to determine significance thresholds for multiple testing correction.

### *In situ* cell phenotyping

Cell phenotyping in each CNS ROI from LACV-infected and mock animals was performed on multiple adjacent FFPE sections (5 μm—thick) by a routine brightfield DAB (brown detection signal) immunohistochemistry with a nuclear counterstaining using hematoxylin (blue). The following primary antibodies were used: mouse monoclonal MAP2 (Millipore; # 05–346; 1:6000), mouse monoclonal PanN (Millipore; # MAB2300; 1:500), mouse monoclonal TH (Millipore; # MAB377; 1:700), polyclonal rabbit GFAP (Dako; # Z 0334; 1:4000), mouse monoclonal CD68 (Dako; Clone KP1; # M0814; 1:500), mouse monoclonal CD4 (Biocare Medical; 4B12; # 3148; 1:25), mouse monoclonal CD8 (Biocare Medical; # CM 154C; 1:50), mouse monoclonal CD20 (Biocare Medical; Clone 126; # CM 004C; 1:400), and mouse monoclonal CD138 (Biocare Medical; Clone B-A38; # 167; 1:10). The ApopTag Plus In Situ kit (Millipore) was used to detect the apoptotic cells according to the manufacturer’s instructions.

### Digital pathology

Immunohistochemically stained tissue sections were scanned at 40x magnification using the ScanScope AT2 (Leica Biosystems). eSlide Manager software was used for digital slide cataloging, storage, and server access. ImageScope software was used for digital slide viewing, analysis, and ROI image acquisition, all complemented by the primate brain maps [27] for precise neuroanatomical definitions of the CNS ROIs and their selections for RNA-seq and molecular pathology. The digital pathology workflow was as follows: (1) an entire stained FFPE tissue section was first analyzed at low magnification; (2) when present, focal pathological inflammatory areas were identified at increasing magnifications using nuclear hematoxylin counterstaining as areas of perivascular/pial/parenchymal hypercellularity; (3) identified inflammatory foci were confirmed by analyzing sequential tissue sections stained with the markers for microglia/macrophages (CD68) and lymphocytes (CD4, CD8, and CD20); (4) inflammatory foci were further interrogated by the immunostaining for astrocytes (GFAP) and several neuronal markers (MAP2, PanN, and TH) to identify specific alterations in the cellular compartments of neurons (i.e., somata, dendrites, axons, and synapses) at high magnification (40x); (5) high magnification representative images of immunoreactivity for each protein marker were used to create figure panels to show the LAVC-infection associated changes compared to dpi-matched mock side-by-side.

## Supporting information

S1 Fig Overview of the CNS regions of interest (ROIs) analyzed in this study by molecular pathology and RNA-seq. (**A—D**) Digital pathology scans show overview low magnification (1x) images of representative whole FFPE tissue sections containing indicated CNS ROIs that were analyzed in this study by RNA-seq and/or molecular pathology according to the study design shown in Fig 1A and 1B). (**A**—**E**) Shown is the representative immunohistochemical staining for the microtubule associated protein 2 (MAP2; brown) with hematoxylin (H; blue) counterstaining in the CNS ROIs of two NHPs at 14 dpi after LACV inoculation (NHPs are indicated as #1 or #2). MAP2 immunoreactivity (MAP2-IR) clearly reveals the ROIs by delineating the gray matter (containing neuronal somatodendritic compartments) from the white matter (contains neuronal axons, devoid MAP2-IR, and appears light blue). Dashed lines in **A** indicate the midline between the telencephalon hemispheres (a portion of the contralateral hemisphere adjacent to the midline was dissected and included to avoid the tissue distortion during formalin fixation by preserving the midline structures). The basal ganglia (**A**, FFPE section on the right) are comprised of the caudate nucleus (CdN), globus pallidus (GP), and putamen (Pu). The section also contains the hippocampus (Hi) and partially temporal cortex (TCx). (**B** and **C**) The cerebellum and brainstem (pons and medulla oblongata) were dissected sagittaly to include a maximum tissue representation in one paraffin block. For RNA-seq, the cerebellum and brainstem were divided along the dashed line separating B and C and collected separately. (**D**) Six FFPE samples at three levels of the spinal cord (two from each of the cervical [C], thoracic [T], and lumbar [L] regions) were embedded into a single paraffin block to maximize tissue representation in each FFPE section for the downstream molecular pathology and RNA-seq analyses. All shown CNS ROIs were analyzed for each of two LACV-infected NHPs and one mock NHP at each time point after inoculation (3, 7, 14, and 21 dpi) by RNA-seq and digital pathology. Additional CNS regions analyzed by digital pathology included portions of the cerebral cortex (**E**) located posteriorly (parietal cortex, PCx) and laterally (temporal cortex, TCx) to the coronal levels shown in A and the midbrain (SN, substantia nigra, dopaminergic neurons revealed by TH [tyrosine hydroxylase] immunoreactivity [brown with blue H counterstain) (**F**). Yellow circles in A and B overlay visible inflammatory foci (inflammatory perivascular/parenchymal/pial infiltration/hypercellularity revealed by the blue H nuclear hematoxylin counterstain) that were further analyzed in detail at increasing magnifications by molecular pathology. Scale bars: 1 mm (A—F).
(TIF)

S2 Fig Spatiotemporal detection of infectious LACV in the CNS of individual NHPs. (**A—C**) Bar graphs show mean LACV titers (+SE) at indicated dpi from 1–4 fresh tissue samples from each of 11 CNS ROIs of individual LACV-infected NHPs that were transcardially perfused with saline to remove any potential virus input from the blood and one CSF sample collected before necropsy from each individual NHP. The limit of virus detection (LOD; 1.7 log_10_ PFU/g) is indicated by the gray dashed lines. P values in each graph indicate that the differences in distribution of LACV titers across the CSF and CNS parenchyma samples were not statistically significant (P > 0.05) between two NHPs at each dpi (two-tailed unequal variance TTEST).
(TIF)

S3 Fig Supporting information for transient focal changes in the somatodendritic compartments of the cortical neurons of LACV-infected NHPs. (**A**—**H**) MAP2 immunoreactivity (MAP2-IR, brown with blue counterstaining) in the cerebral cortex is shown for each of two LACV-infected NHPs (NHP #1 and NHP #2) at indicated dpi. A panel for each NHP contains three overview images (1–3) with an increasing magnification (1x, 5x, and 20x, respectively) in support to high-magnification images shown in Fig 2B. The boxed areas in 1x magnification images (A1 –H1) are shown in the 5x magnification images (A2 –H2) and boxed areas in 5x magnification images (A2 –H2) are shown in the 20x magnification images (A3 –H3). The scale bars are provided for each image. Note focal non-uniform dendritic MAP2-IR in association with various degrees of perivascular lymphocytic infiltration at 7 and 14 dpi (C—F), compared to a normal appearance of the somatodendritic MAP2-IR at 3 dpi (A and B) and 21 dpi (G and H).
(TIF)

S4 Fig Supporting information for corresponding changes in protein immunoreactivities in dpi matched LACV-infected NHPs. **(A—P)** Shown are the corresponding representative tissue fields for the indicated immunoreactivities (brown with blue counterstaining; rows) during LACV infection at 3, 7, 14, and 21 dpi (columns) for the dpi matched NHPs other than those shown in the figures in the text. (**A**—**D**) PanN-IR corresponding to shown in Fig 2D. (**E**—**H**) TH-IR corresponding to shown in Fig 2E and 2F. The focal non-uniform TH-IR and loss of TH-IR in the somata of substantia nigra neurons was observed only at 7 dpi and shown by inset in F. (**I**—**L**) GFAP-IR corresponding to shown in Fig 5E–5H. (**M**—**P**) CD68-IR corresponding to shown in Fig 7C. Scale bars: 50 μm.
(TIF)

S5 Fig Time-limited downregulation of cerebellar neurophysiological processes in NHPs during LACV infection. (**A**) A temporal heatmap shows functional enrichment for transcriptional downregulation of the neurophysiological processes during 3 weeks after LACV inoculation. The color scale is based on the significance (-log_10_ p-adj). The gene ontology (GO) sources: BP, Biological Process; CC, Cellular Component; and MF, Molecular Function (terms are highlighted by respective colors). Lists of genes downregulated in the cerebellum at each dpi and data associated with (A) are provided in S1 File. (**B**—**I**) Visualization of the temporal changes in MAP2 protein expression (MAP2 immunoreactivity, MAP2-IR, brown with blue counterstaining) in the neuronal somatodendritic compartments of the cerebellar cortex in the dpi-matched mock (**B**—**E**) and representative areas in LACV-inoculated primates (**F—I**). Note: (i) pial lymphocytic infiltration (yellow arrows) appeared at 7 dpi (**G**), increased at 14 dpi (**H**), and decreased at 21 dpi (returning to the level seen at 7 dpi) (**I**); (ii) a rare focal lesion (red asterisk in H) with depleted MAP2-IR in the Purkinje cell layer (PCL), granule cell layer (GrCL) neurons, and partially in the molecular layer (ML) is spatially associated with the increased pial lymphocytic infiltration. The labeling keys for pathological changes in F—I are indicated below the panels. Scale bars: 50 μm (B—I).
(TIF)

S6 Fig Neurophysiological processes in the pons/medulla (brainstem) and spinal cord of NHPs are maintained at normal levels during LACV infection. (**A**) Temporal heatmaps show functional enrichment for transcriptional downregulation during 3 weeks after LACV inoculation. The color scale is based on the significance (-log_10_ p-adj). The gene ontology (GO) sources: BP, Biological Process; CC, Cellular Component; and MF, Molecular Function (terms are highlighted by respective colors). Lists of genes downregulated in the brainstem and spinal cord at each dpi and data associated with (A) are provided in S1 File. (**B—M**) Representative MAP2-IR (brown with blue counterstaining) in the somatodendritic compartments of the pons (pontine nuclei) (**B**), medulla oblongata (inferior olives; IO) (**C**), and spinal cord (cervical) (**D—M**) is shown at indicated dpi after LACV inoculation, compared to mock (3 dpi) as normal reference. Note a slight hypercellularity (increased number of the blue [hematoxylin-counterstained] nuclei) in the neuropil of the pons (B; 7 dpi panel), medulla (C; 7 dpi panel), and spinal cord (L; 14 dpi panel) in LACV-infected NHPs. (**I—M**) Respective high magnification fields of the ventral horn areas circled in the overviews of the entire transverse spinal cord sections (**D—H**) show the spinal motor neurons and surrounding neuropil. Scale bars: 50 μm (B, C, I—M); 500 μm (D—H).
(TIF)

S7 Fig CD68+ CNS-border macrophages in the telencephalon of mock-inoculated NHPs. **(A—C)** Representative CD68-IR visualizes the CNS-border macrophages in the pia mater, subpial parenchyma, deeper cerebral cortex parenchyma, and basal ganglia at indicated dpi. The cell labeling keys are referenced in the bottom of the figure. Each row documents appearance of the indicated CD68+ pial and perivascular cells (brown with blue counterstaining) during the time points that match the examined dpi during LACV infection. Note a near absence of CD68-IR in the surrounding parenchyma since surveying microglial cells that reside in the parenchyma do not express high levels of CD68-IR under normal physiological conditions, distinguishing these cells from shown CNS-border macrophages. Scale bars: 50 μm.
(TIF)

S8 Fig Regulation of the phagocytic environment in the cerebellum, brainstem, and spinal cord during LACV infection of the CNS in NHPs. **(A—C)** Representative CD68-IR shows the reactive microglial cells and CNS-border macrophages in indicated CNS ROIs. The cell labeling keys are referenced in the top left, followed by the color keys used to highlight the specific panels representing stages in regulation of the phagocytic environment. Spinal motor neurons (SMNs) with the satellite CD68+ microglia are outlined in cyan in C. Each row compares morphological changes in the indicated CD68+ cells (brown with blue counterstaining) during LACV infection (dpi indicated) and mock (14 dpi shown as a reference for the peak changes in LACV). Scale bars: 50 μm.
(TIF)

S9 Fig Spatiotemporal clustering analysis of gene expression data from individual LACV-infected NHPs. (**A**—**D**) Dendrograms show the results of average linkage clustering (Pearson) by displaying the distances (or similarities) (x-axis) between gene expression (transformed reads count matrix [dseq2]) in indicated CNS ROIs of individual LACV-infected NHPs (NHP #1 and NHP #2) at each indicated dpi (right y-axis). The clusters that are closest together (most similar) are shown in bold black and the second-order agglomerative clustering is shown in gray. Note the grouping of the biological replicates (NHP #1 and NHP #2) at each time point, indicating a high similarity in their gene expression.
(TIF)

S1 Table Virus neutralizing antibodies in the serum and cerebrospinal fluid of NHPs after intrathalamic inoculation with LACV. (DOCX)

S1 File Lists of genes downregulated in each CNS ROI at each dpi and data associated with Figs 2A, S5A and S6A. (XLSX)

S2 File Lists of genes upregulated in each CNS ROI at each dpi and data associated with Fig 3. (XLSX)

S3 File Lists of time-differentially expressed genes in each CNS ROI and data associated with Fig 4. (XLSX)
