# Supplementary figures and images for: Spatiotemporal profile of an optimal host response to virus infection in the primate central nervous system
Source: PLoS Pathog. 2025 Jan 22;21(1):e1012530. doi: 10.1371/journal.ppat.1012530 (PMC11753669; doi:10.1371/journal.ppat.1012530)

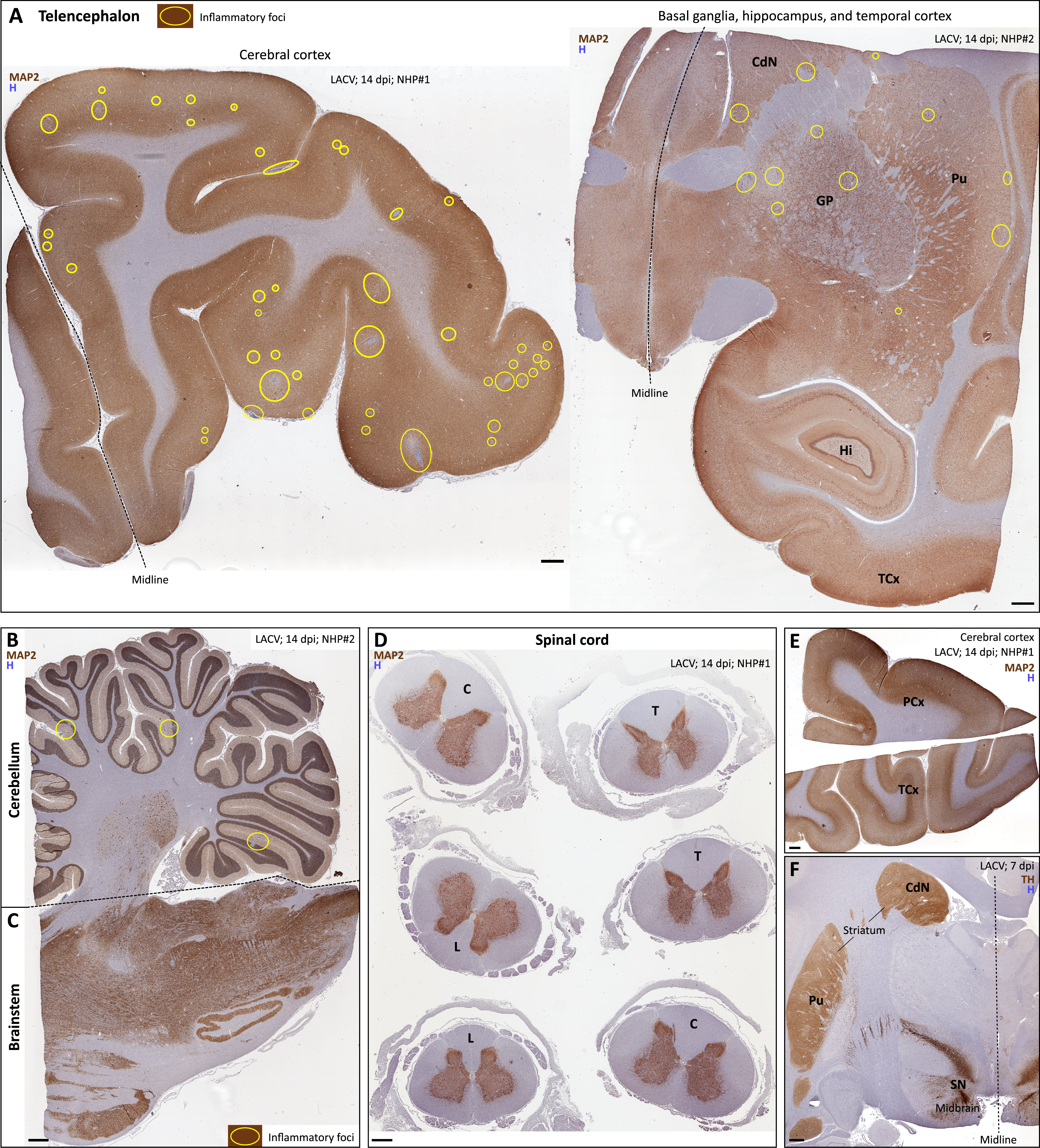

Supplement: S1 Fig — (A—D) Digital pathology scans show overview low magnification (1x) images of representative whole FFPE tissue sections containing indicated CNS ROIs that were analyzed in this study by RNA-seq and/or molecular pathology according to the study design shown in Fig 1A and 1B). (A—E) Shown is the representative immunohistochemical staining for the microtubule associated protein 2 (MAP2; brown) with hematoxylin (H; blue) counterstaining in the CNS ROIs of two NHPs at 14 dpi after LACV inoculation (NHPs are indicated as #1 or #2). MAP2 immunoreactivity (MAP2-IR) clearly reveals the ROIs by delineating the gray matter (containing neuronal somatodendritic compartments) from the white matter (contains neuronal axons, devoid MAP2-IR, and appears light blue). Dashed lines in A indicate the midline between the telencephalon hemispheres (a portion of the contralateral hemisphere adjacent to the midline was dissected and included to avoid the tissue distortion during formalin fixation by preserving the midline structures). The basal ganglia (A, FFPE section on the right) are comprised of the caudate nucleus (CdN), globus pallidus (GP), and putamen (Pu). The section also contains the hippocampus (Hi) and partially temporal cortex (TCx). (B and C) The cerebellum and brainstem (pons and medulla oblongata) were dissected sagittaly to include a maximum tissue representation in one paraffin block. For RNA-seq, the cerebellum and brainstem were divided along the dashed line separating B and C and collected separately. (D) Six FFPE samples at three levels of the spinal cord (two from each of the cervical [C], thoracic [T], and lumbar [L] regions) were embedded into a single paraffin block to maximize tissue representation in each FFPE section for the downstream molecular pathology and RNA-seq analyses. All shown CNS ROIs were analyzed for each of two LACV-infected NHPs and one mock NHP at each time point after inoculation (3, 7, 14, and 21 dpi) by RNA-seq and digital pathology. Additiona [file ppat.1012530.s001.tif]

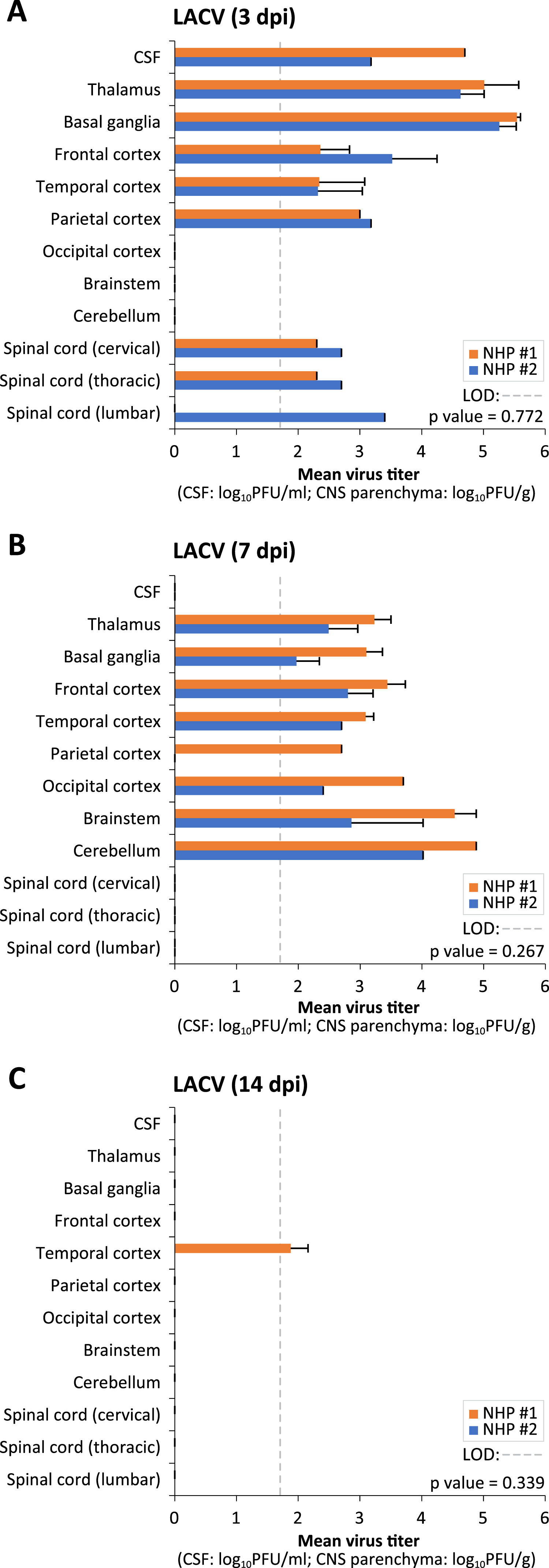

Supplement: S2 Fig — (A—C) Bar graphs show mean LACV titers (+SE) at indicated dpi from 1–4 fresh tissue samples from each of 11 CNS ROIs of individual LACV-infected NHPs that were transcardially perfused with saline to remove any potential virus input from the blood and one CSF sample collected before necropsy from each individual NHP. The limit of virus detection (LOD; 1.7 log10 PFU/g) is indicated by the gray dashed lines. P values in each graph indicate that the differences in distribution of LACV titers across the CSF and CNS parenchyma samples were not statistically significant (P > 0.05) between two NHPs at each dpi (two-tailed unequal variance TTEST). (TIF) [file ppat.1012530.s002.tif]

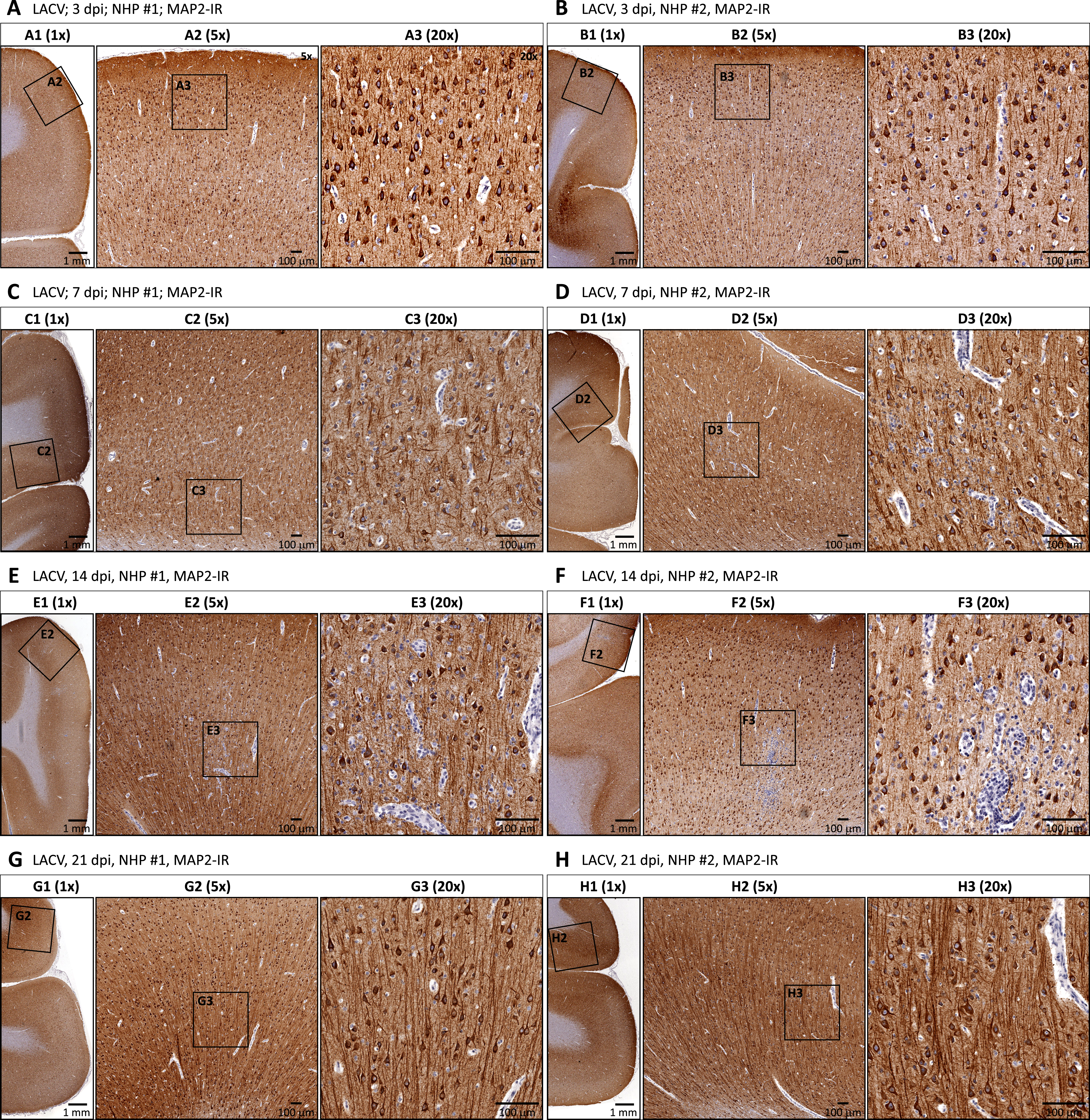

Supplement: S3 Fig — (A—H) MAP2 immunoreactivity (MAP2-IR, brown with blue counterstaining) in the cerebral cortex is shown for each of two LACV-infected NHPs (NHP #1 and NHP #2) at indicated dpi. A panel for each NHP contains three overview images (1–3) with an increasing magnification (1x, 5x, and 20x, respectively) in support to high-magnification images shown in Fig 2B. The boxed areas in 1x magnification images (A1 –H1) are shown in the 5x magnification images (A2 –H2) and boxed areas in 5x magnification images (A2 –H2) are shown in the 20x magnification images (A3 –H3). The scale bars are provided for each image. Note focal non-uniform dendritic MAP2-IR in association with various degrees of perivascular lymphocytic infiltration at 7 and 14 dpi (C—F), compared to a normal appearance of the somatodendritic MAP2-IR at 3 dpi (A and B) and 21 dpi (G and H). (TIF) [file ppat.1012530.s003.tif]

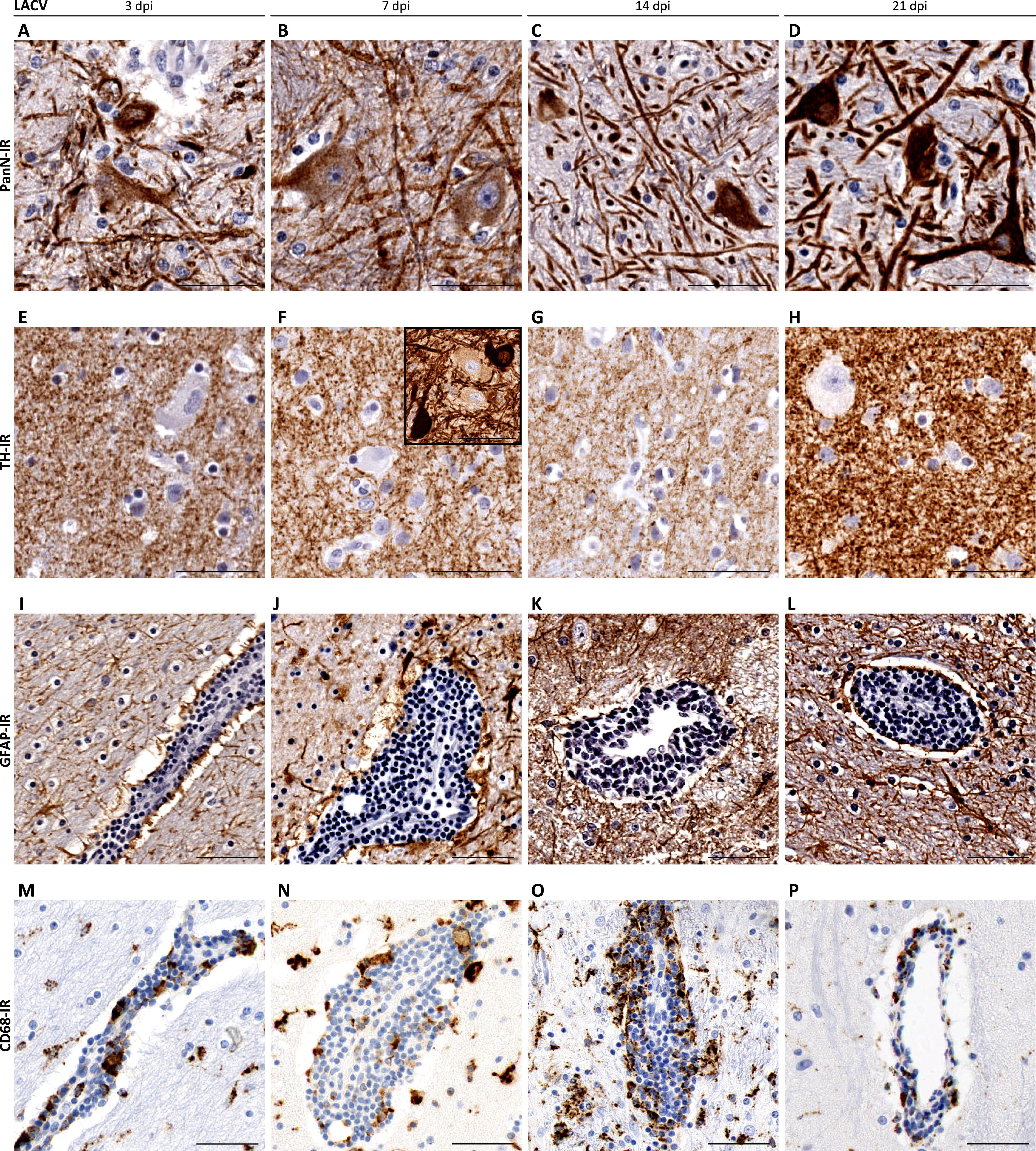

Supplement: S4 Fig — (A—P) Shown are the corresponding representative tissue fields for the indicated immunoreactivities (brown with blue counterstaining; rows) during LACV infection at 3, 7, 14, and 21 dpi (columns) for the dpi matched NHPs other than those shown in the figures in the text. (A—D) PanN-IR corresponding to shown in Fig 2D. (E—H) TH-IR corresponding to shown in Fig 2E and 2F. The focal non-uniform TH-IR and loss of TH-IR in the somata of substantia nigra neurons was observed only at 7 dpi and shown by inset in F. (I—L) GFAP-IR corresponding to shown in Fig 5E–5H. (M—P) CD68-IR corresponding to shown in Fig 7C. Scale bars: 50 μm. (TIF) [file ppat.1012530.s004.tif]

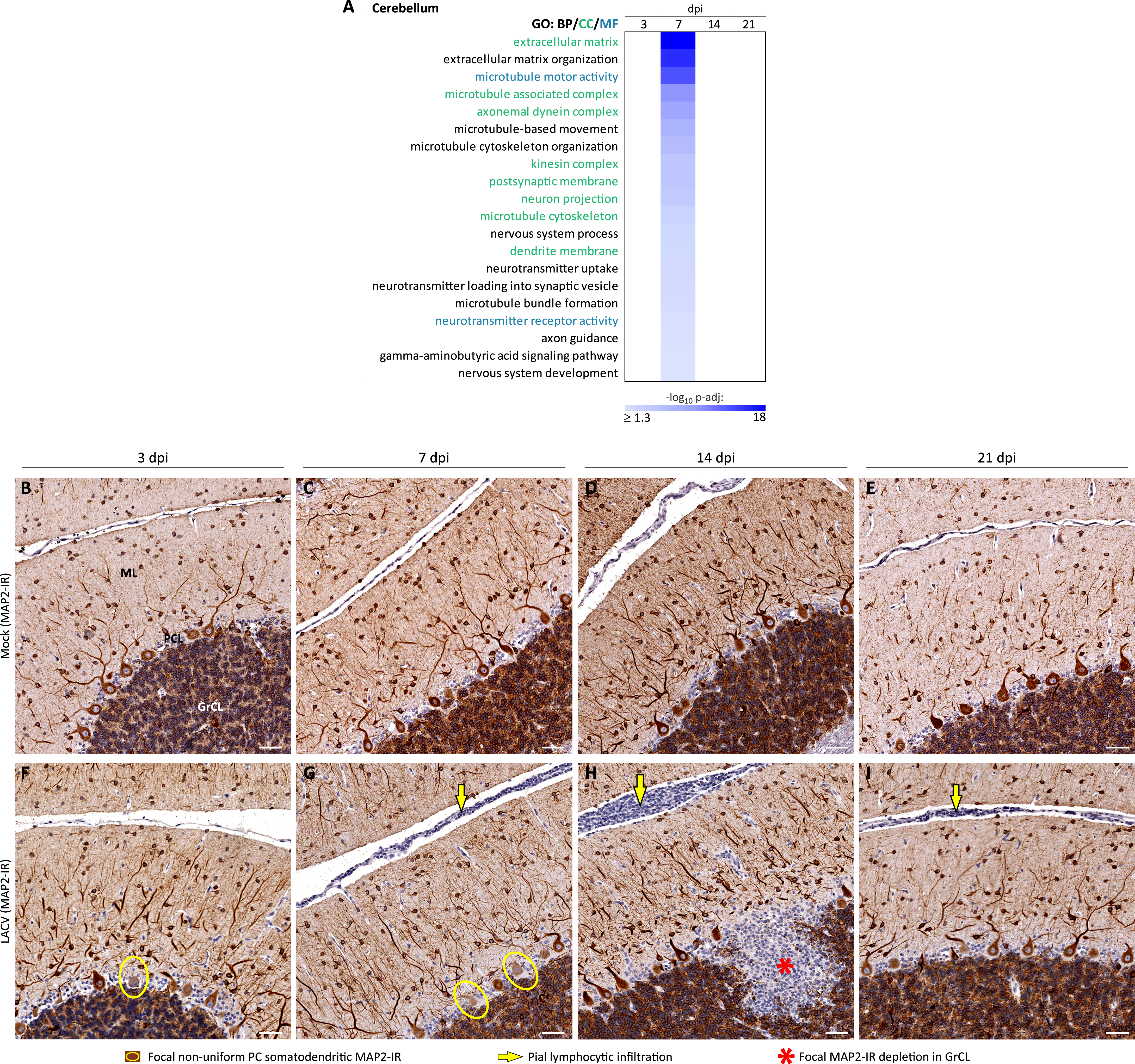

Supplement: S5 Fig — (A) A temporal heatmap shows functional enrichment for transcriptional downregulation of the neurophysiological processes during 3 weeks after LACV inoculation. The color scale is based on the significance (-log10 p-adj). The gene ontology (GO) sources: BP, Biological Process; CC, Cellular Component; and MF, Molecular Function (terms are highlighted by respective colors). Lists of genes downregulated in the cerebellum at each dpi and data associated with (A) are provided in S1 File. (B—I) Visualization of the temporal changes in MAP2 protein expression (MAP2 immunoreactivity, MAP2-IR, brown with blue counterstaining) in the neuronal somatodendritic compartments of the cerebellar cortex in the dpi-matched mock (B—E) and representative areas in LACV-inoculated primates (F—I). Note: (i) pial lymphocytic infiltration (yellow arrows) appeared at 7 dpi (G), increased at 14 dpi (H), and decreased at 21 dpi (returning to the level seen at 7 dpi) (I); (ii) a rare focal lesion (red asterisk in H) with depleted MAP2-IR in the Purkinje cell layer (PCL), granule cell layer (GrCL) neurons, and partially in the molecular layer (ML) is spatially associated with the increased pial lymphocytic infiltration. The labeling keys for pathological changes in F—I are indicated below the panels. Scale bars: 50 μm (B—I). (TIF) [file ppat.1012530.s005.tif]

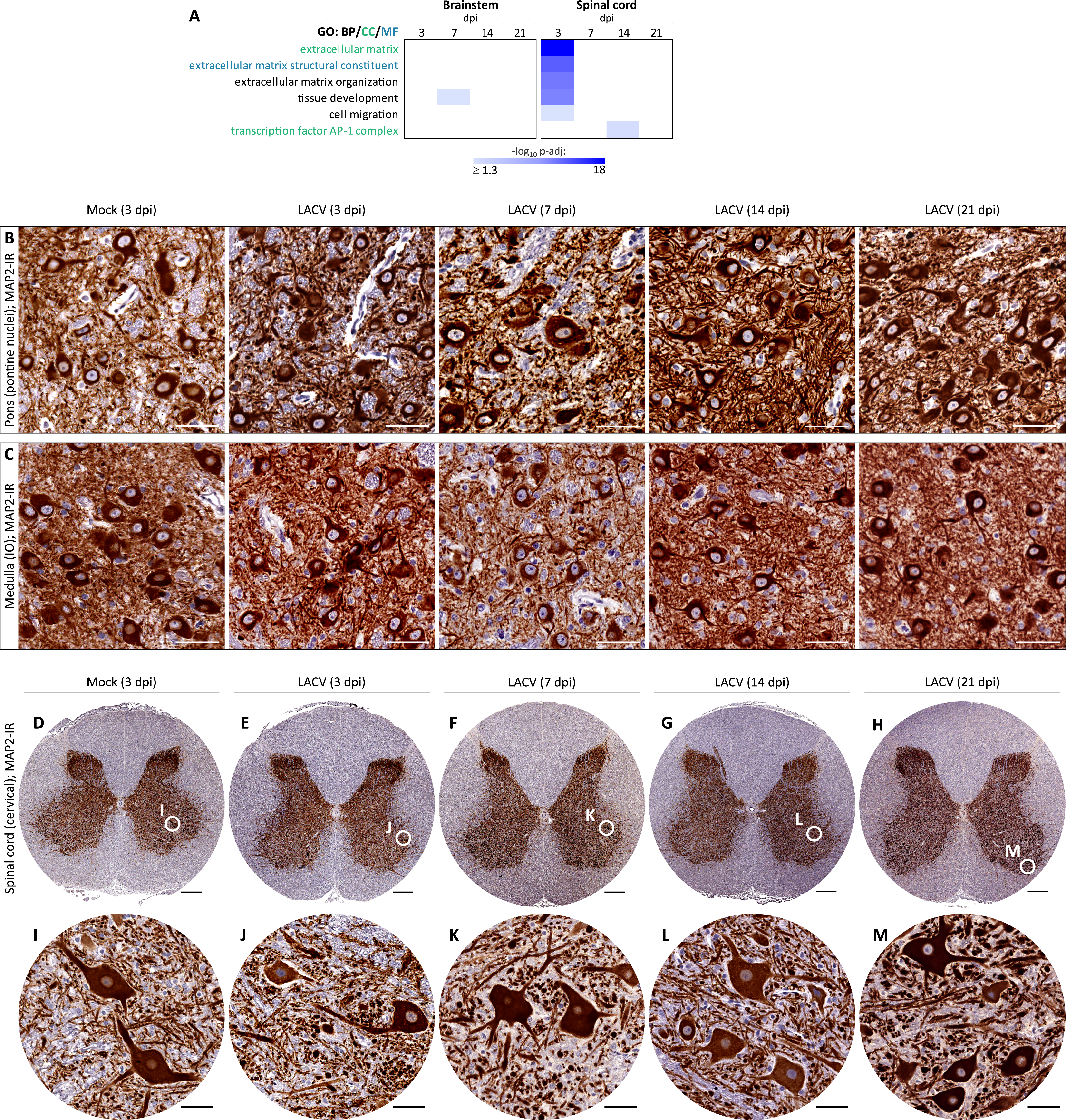

Supplement: S6 Fig — (A) Temporal heatmaps show functional enrichment for transcriptional downregulation during 3 weeks after LACV inoculation. The color scale is based on the significance (-log10 p-adj). The gene ontology (GO) sources: BP, Biological Process; CC, Cellular Component; and MF, Molecular Function (terms are highlighted by respective colors). Lists of genes downregulated in the brainstem and spinal cord at each dpi and data associated with (A) are provided in S1 File. (B—M) Representative MAP2-IR (brown with blue counterstaining) in the somatodendritic compartments of the pons (pontine nuclei) (B), medulla oblongata (inferior olives; IO) (C), and spinal cord (cervical) (D—M) is shown at indicated dpi after LACV inoculation, compared to mock (3 dpi) as normal reference. Note a slight hypercellularity (increased number of the blue [hematoxylin-counterstained] nuclei) in the neuropil of the pons (B; 7 dpi panel), medulla (C; 7 dpi panel), and spinal cord (L; 14 dpi panel) in LACV-infected NHPs. (I—M) Respective high magnification fields of the ventral horn areas circled in the overviews of the entire transverse spinal cord sections (D—H) show the spinal motor neurons and surrounding neuropil. Scale bars: 50 μm (B, C, I—M); 500 μm (D—H). (TIF) [file ppat.1012530.s006.tif]

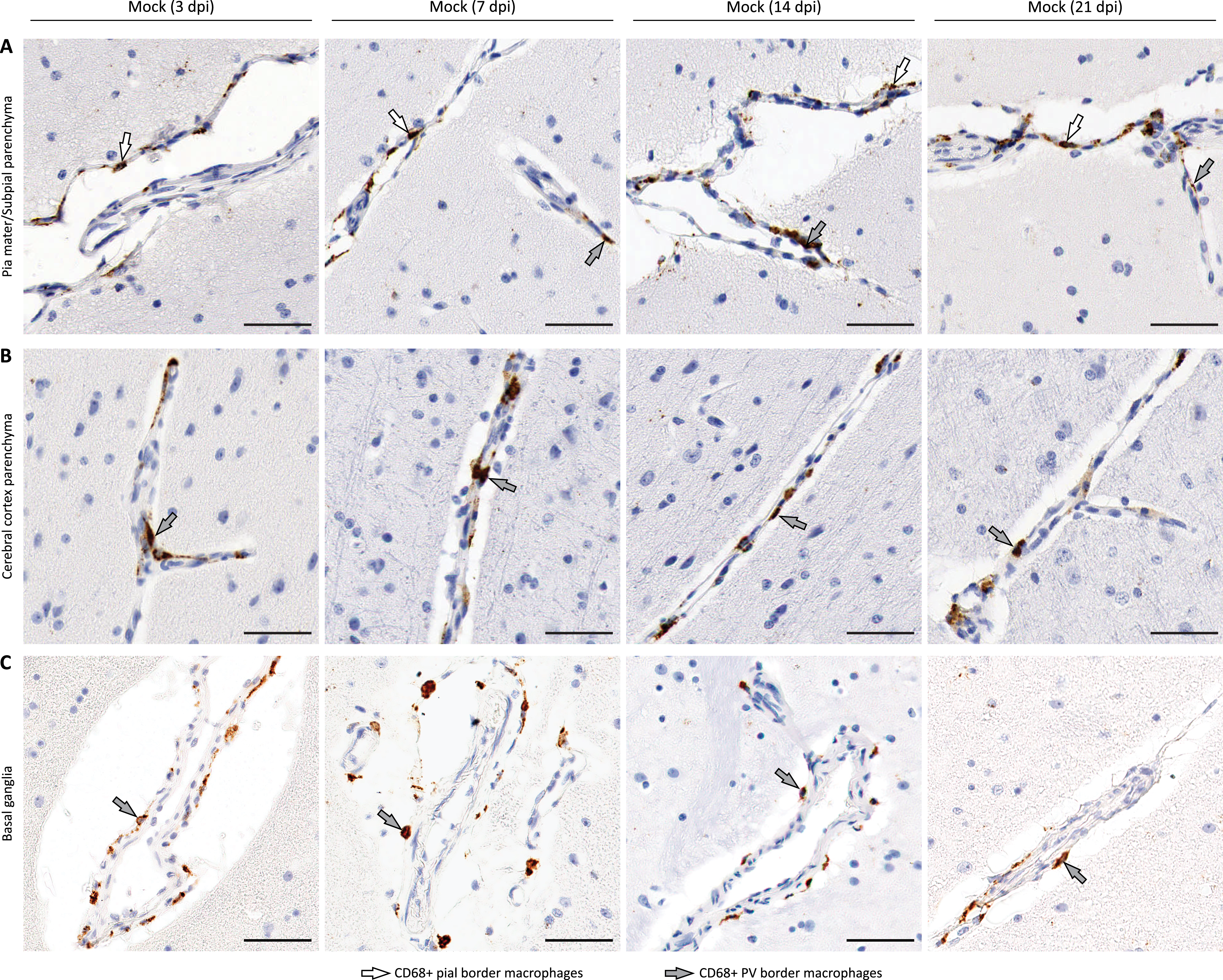

Supplement: S7 Fig — (A—C) Representative CD68-IR visualizes the CNS-border macrophages in the pia mater, subpial parenchyma, deeper cerebral cortex parenchyma, and basal ganglia at indicated dpi. The cell labeling keys are referenced in the bottom of the figure. Each row documents appearance of the indicated CD68+ pial and perivascular cells (brown with blue counterstaining) during the time points that match the examined dpi during LACV infection. Note a near absence of CD68-IR in the surrounding parenchyma since surveying microglial cells that reside in the parenchyma do not express high levels of CD68-IR under normal physiological conditions, distinguishing these cells from shown CNS-border macrophages. Scale bars: 50 μm. (TIF) [file ppat.1012530.s007.tif]

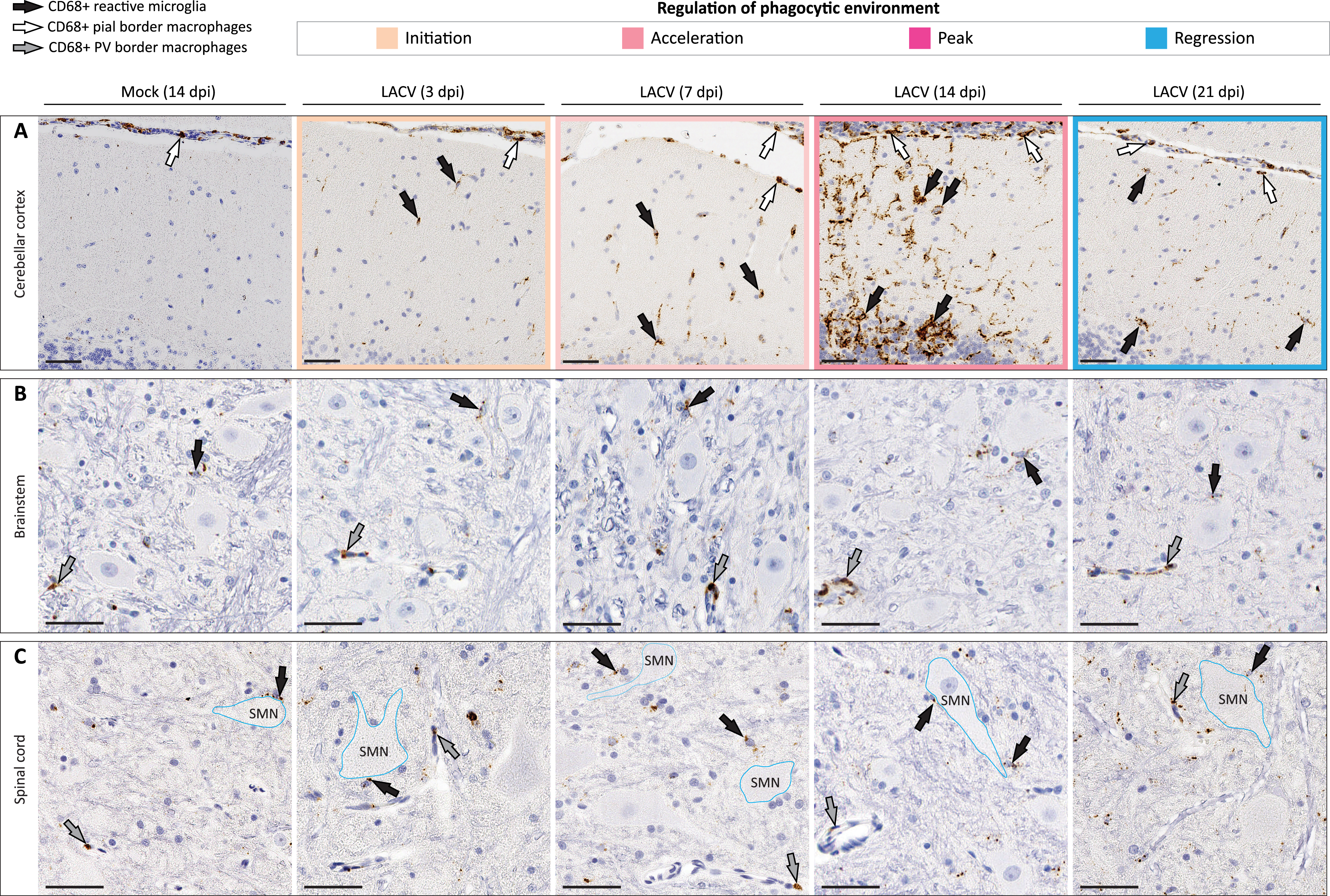

Supplement: S8 Fig — (A—C) Representative CD68-IR shows the reactive microglial cells and CNS-border macrophages in indicated CNS ROIs. The cell labeling keys are referenced in the top left, followed by the color keys used to highlight the specific panels representing stages in regulation of the phagocytic environment. Spinal motor neurons (SMNs) with the satellite CD68+ microglia are outlined in cyan in C. Each row compares morphological changes in the indicated CD68+ cells (brown with blue counterstaining) during LACV infection (dpi indicated) and mock (14 dpi shown as a reference for the peak changes in LACV). Scale bars: 50 μm. (TIF) [file ppat.1012530.s008.tif]

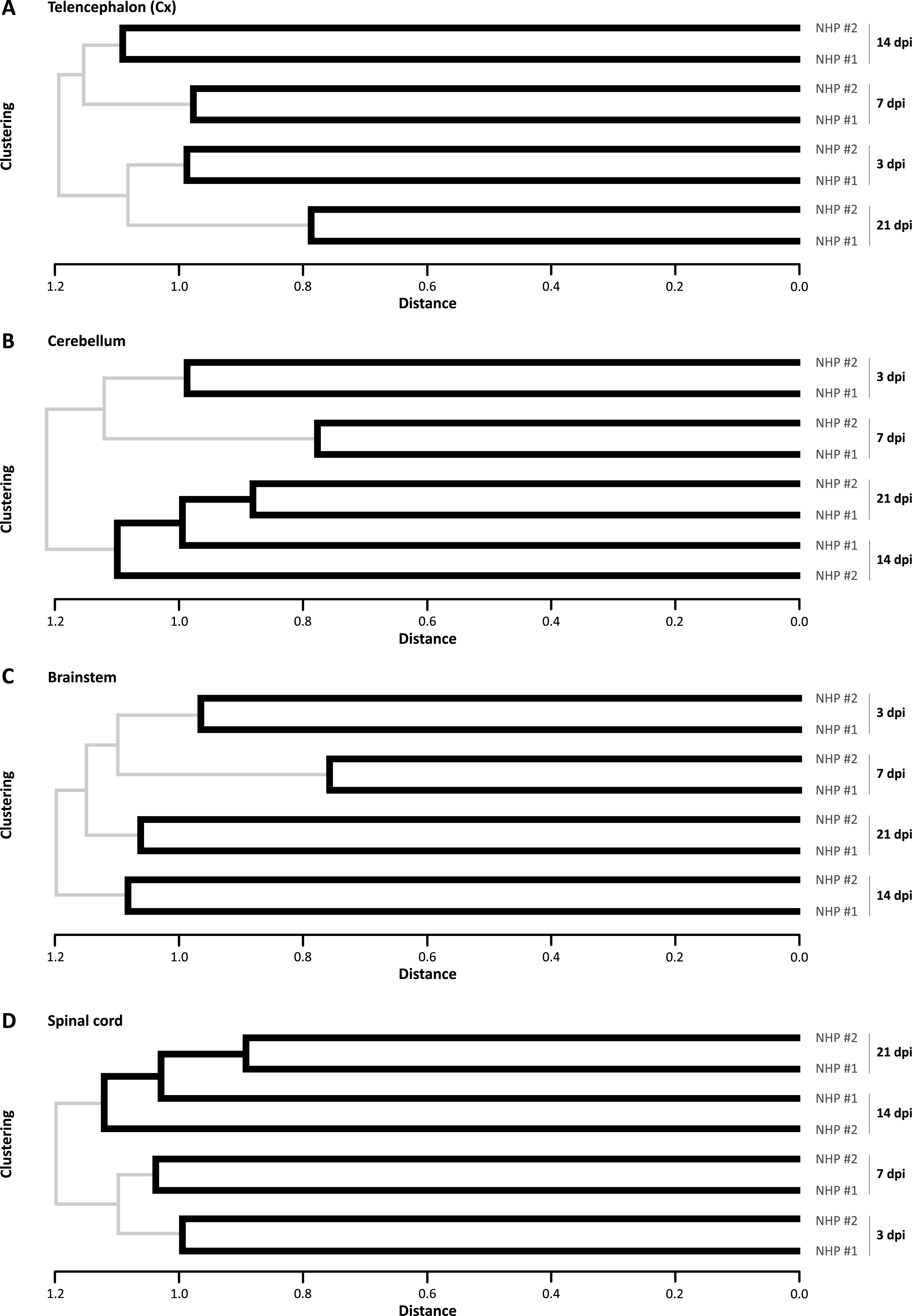

Supplement: S9 Fig — (A—D) Dendrograms show the results of average linkage clustering (Pearson) by displaying the distances (or similarities) (x-axis) between gene expression (transformed reads count matrix [dseq2]) in indicated CNS ROIs of individual LACV-infected NHPs (NHP #1 and NHP #2) at each indicated dpi (right y-axis). The clusters that are closest together (most similar) are shown in bold black and the second-order agglomerative clustering is shown in gray. Note the grouping of the biological replicates (NHP #1 and NHP #2) at each time point, indicating a high similarity in their gene expression. (TIF) [file ppat.1012530.s009.tif]
