# Supplementary material for: Spatiotemporal profile of an optimal host response to virus infection in the primate central nervous system
Source: PLoS Pathog. 2025 Jan 22;21(1):e1012530. doi: 10.1371/journal.ppat.1012530 (PMC11753669; doi:10.1371/journal.ppat.1012530)
Supplement: S1 Table — (DOCX) [file ppat.1012530.s010.docx]

**Table S1. Virus neutralizing antibodies in the serum and cerebrospinal fluid of NHPs after intrathalamic inoculation with LACV.**

| **Inoculum** |  | **Sample type** | **Geometric mean titer for neutralizing antibodies^a^** | | | | | | |
| --- | --- | --- | --- | --- | --- | --- | --- | --- | --- |
|  |  |  | **0 dpi** | **3 dpi** | **5 dpi** | **7 dpi** | **10 dpi** | **14 dpi** | **21 dpi** |
| LACV |  | Serum | <10 | <10 | <10 | <10 | **65** | **409** | **2391** |
|  |  | CSF | nd | <10 | nd | <10 | nd | <10 | **50** |
| Mock |  | Serum | <10 | <10 | 11 | <10 | <10 | <10 | <10 |
|  |  | CSF | nd | <10 | nd | <10 | nd | <10 | <10 |

^a^ Reciprocal PRNT_60_. Limit of detection: 1:10 dilution.

Titers above the limit of detection are shaded in gray.

nd – not done (CSF was only collected at the time of necropsy).
